# Long-Lived Charge Carrier Photogeneration in a Cooperative Supramolecular Double-Cable Polymer

**DOI:** 10.1021/jacs.4c09637

**Published:** 2024-10-22

**Authors:** Jan Joseph, José Augusto Berrocal, Nicolás M. Casellas, Dirk M. Guldi, Tomás Torres, Miguel García-Iglesias

**Affiliations:** † Friedrich-Alexander-Universität Erlangen-Nürnberg, FAU Profile Center Solar, Department of Chemistry and Pharmacy, Interdisciplinary Center for Molecular Materials (ICMM), Egerlandstr. 3, 91058 Erlangen, Germany; ‡ Institute of Chemical Research of Catalonia (ICIQ), Barcelona Institute of Science and Technology (BIST), Avda. Països Catalans 16, 43007 Tarragona, Spain; § QUIPRE Department, Nanomedicine-IDIVAL, Universidad de Cantabria, Avd. de Los Castros, 46, 39005 Santander, Spain; ∥ Departamento de Química Orgánica, Universidad Autónoma de Madrid, Cantoblanco, 28049 Madrid, Spain; ⊥ Institute for Advanced Research in Chemical Sciences (IAdChem), Universidad Autonoma de Madrid (UAM), 28049 Madrid, Spain; # IMDEA-Nanociencia, c/Faraday, 9, Cantoblanco, 28049 Madrid, Spain

## Abstract

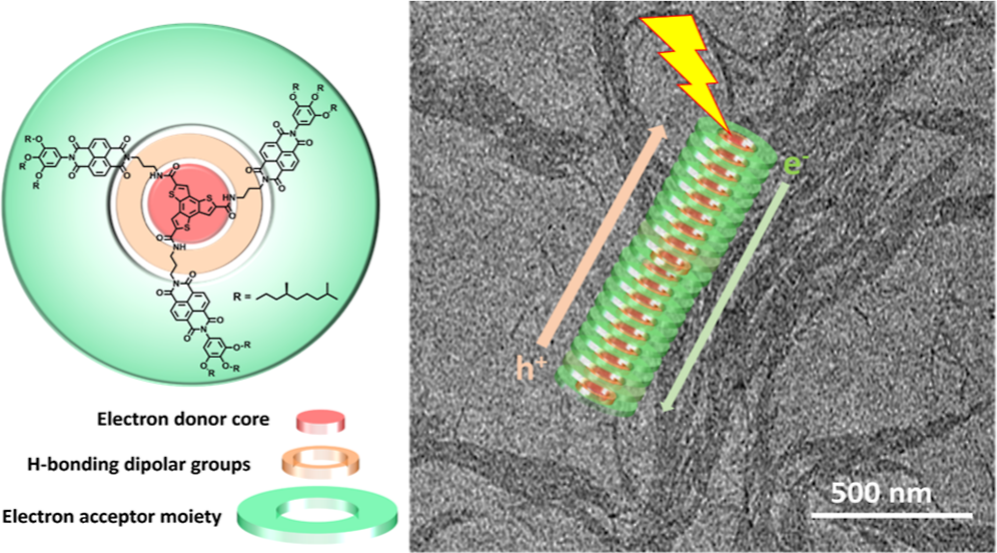

A newly designed C_3_-symmetric disc-shaped chromophore, **BTT(NDI)_3_**, features electron accepting naphthalene diimides linked to an electron donor BTT core. **BTT(NDI)_3_** self-assembles in apolar solvents into highly ordered, chiral supramolecular fibers through π–π and 3-fold hydrogen-bonding interactions. This leads to a cooperative formation of plane-to-plane stacking of BTTs and J-aggregation of the outer NDIs. Such a structure ensures high charge mobility. Only photoexcitation of BTT in the **BTT(NDI)_3_** polymers triggers a unidirectional electron transfer from BTT to NDI and results in (BTT^•+^-NDI^•–^) lifetimes that are by up to 3 orders of magnitude longer compared to (NDI^•+^-NDI^•–^) that is formed upon NDI photoexcitation. A multiphasic decay implies ambipolar pathways for charge carriers, that is, electron and hole delocalization along the respective BTT and NDI stacks. Our supramolecular approach offers potential for developing functional supramolecular polymers with continuous pathways for electrons and holes and, in turn, minimizing charge recombination losses in organic photovoltaic devices.

## Introduction

Organic photovoltaics (OPV) are among the most striking technologies for a clean and sustainable energy production. What stands out is the lightweight, the flexibility, and the low costs of organic materials.^1^ Despite an outstanding growth, especially in the past decade, current OPV devices suffer from substantial energy losses over time.^2^ Control over organization and morphology of, for example, organic materials are expected to overcome this and other bottlenecks.^3,4^

A common approach to prepare optoelectronic materials for photon-energy conversion is to use photosynthetic mimics of covalent electron donor–acceptor (D–A) systems. Such systems benefit from large D–A interfacial areas.^5^ Limitations of covalent organic synthesis, however, arise when attempting to connect larger and complex building blocks and to create efficient percolation pathways to the electrodes.^6,7^

A viable alternative is the molecular self-assembly to hierarchically order monomeric building-blocks at the nano- and mesoscales so that they undergo photoiduced charge separation. It contrasts those strategies that aim at phase separating D- and A-components to realize long-range order in the active layer.^8−12^ All together noncovalent assemblies support a maximum structural complexity at a minimum of synthetic efforts. A leading example is supramolecular polymerization. It enables the precise organization of molecular chromophores into one-dimensional (1-D) aggregates by means of reversible supramolecular interactions.^13^ Particularly relevant is the case of cooperative supramolecular polymers, which ensure the obtainment of larger and highly ordered nanostructures.^14,15^

The modulation of photogenerated excitons in supra-molecular materials is a prerequisite to operate photovoltaic devices. This property can be achieved by creating assemblies in which each domain is interpenetrated and forms a continuous pathway for the free electrons and holes to travel to their respective electrodes. Weak associations of electronically complementary D and A moieties has led to the creation of a number of supramolecular assemblies, in which D- and A-monomers stack on one another in an alternating fashion.^9,16−20^ This particular architecture has produced field effect transistors with excellent properties. However, the performance of similarly structured photovoltaic devices has not been comparable to that of the OFETs due to the instantaneous trapping of the photogenerated charge carriers by geminate charge recombination.^21^ A plausible strategy to overcome this challenge consists in using self-complementary interactions such as hydrogen-bonding. It would minimize the cofacial arrangement of electronically complementary aromatic chromophores, on one hand, and force the monomeric units to stack into columnar aggregates comprising only one monomer, i.e., homodomains, on the other hand.^22,23^ Using directional H-bonding perpendicular to the polymer axis has also been shown to positively affect the long-range transport of light in homopolymers. In addition, it provides structural reinforcement.^24,25^ Self-segregated supramolecular fibers^26^ or block-like supramolecular copolymers^27^ have displayed major drawbacks in photovoltaic applications that can be attributed to the limited surface areas between Ds and As.

In an alternative approach, covalent and noncovalent means are integrated together. To this end, Ds and As are covalently linked to afford a single monomer followed by supramolecular interactions such as π–π interactions and solvophobic effects,^28−30^ or hydrogen bonding antiparallel^31−33^ and, more recently, parallel to the fiber axis.^34−37^ All of the aforementioned have provided both, large coaxial D/A heterojunctions and continuous pathways for electrons and holes to transverse to their respective electrodes. The electron-rich/-poor bicontinuous networks generated in the resulting “double-cable” structures effectively prolong the lifetimes of photogenerated charge carriers. It is mainly the segregated domains of the D- and A-counterparts that generate ambipolar pathways for the charge carriers.^28−37^

In this context, thiophene-based π-conjugated organic materials have raised considerable interest due to their use as electron donating organic semiconductors.^38^ Particularly relevant for this work, the planarized star-shaped structure of benzotrithiophene (BTTs) allows to create columnar plane-to-plane stacks by a combination of π–π interactions and hydrogen bonds.^39^ BTTs supra-molecular assemblies have shown excellent, quasi-temperature independent, hole-transporting properties,^40−42^ and are capable of controlling the electron/hole transport along the columnar axis using the polarization of the head-to-tail hydrogen bonding arrays parallel to the columnar axis.^43^ A perfect match for n-type BTTs are p-type naphthalenediimides (NDIs). They exhibit excellent charge carrier mobilities in field effect transistors,^44^ a property that has been used to build a range of supramolecular materials in combination with other p-conjugated molecules.^9,16−22^ Recent work has also highlighted that the lifetimes of photogenerated charge-carriers are significantly prolonged in J-aggregates of NDIs through delocalization.^45,46^

**Scheme 1:**
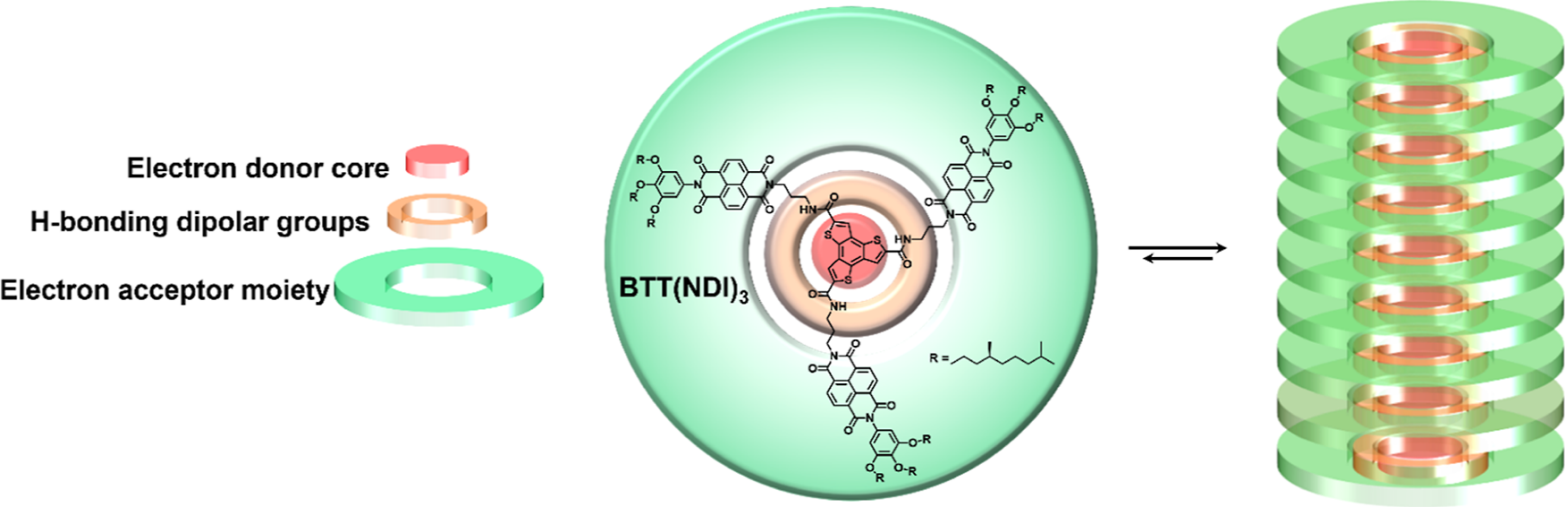
Chemical Structure of the **BTT(NDI)_3_** Disc (Left) and Schematic Representation of Its Self-Assembly Into Helical Nanostructures (Right)

By means of combining the features of BTTs and NDIs, we report on a newly designed C_3_-symmetric disc shaped chromophore comprising NDIs anchored onto a BTT core **(BTT(NDI)_3_**; Scheme 1). In our design, the electronically complementary Ds and As are covalently linked to each other. Their close proximity powers an efficient charge separation and avoids complications associated with either phase segregation or long exciton diffusion lengths. A cooperative mechanism is operative by which **BTT(NDI)_3_** discotics self-assemble into one-dimensional nanofibers in apolar solvents. The self-assembly process is controlled by a combination of π–π and 3-fold hydrogen-bonding interactions that force the BTT and NDI subunits to homostack. While the BTT units stack in a plane-to-plane fashion, the outer NDI units form J-aggregates due to the helical nature of the 1-D fibers. Such a unique configuration ensures that, upon photoexcitation, charges migrate through electron-rich and -poor bicontinuous networks and that charge recombination is significantly delayed.

## Results and Discussion

### Monomer Design

The molecular design of the self-assembling **BTT(NDI)**_**3**_ unit is based on a C_3_-symmetric benzotrithiophene tricarboxamide (BTTTA) semiconducting supramolecular 1D-polymer previously reported by us.^43^ BTTTA is derived from an electron donating BTT^40−42^ core and is covalently linked to electronically complementary, electron accepting NDIs^44^ via amide bonds (Scheme 1). The three amide moieties in the **BTT(NDI)**_**3**_ design support 3-fold head to-tail hydrogen bonds between discotics in apolar solvents. These directional hydrogen bonding interactions force the monomeric units to homostack into columnar aggregates. The NDI unsymmetrical chromophores are connected to the BTTTA core by a short aliphatic spacer to introduce some flexibility in the **BTT(NDI)**_**3**_ monomer. Further, all NDIs are equipped with (*S*)-(−)-3,7-dimethyloctyloxy side chains at their periphery to bias the chirality of the helical supramolecular polymer. Overall, the **BTT(NDI)**_**3**_ design is expected to force Ds and As into close proximity to each other. This per se is expected to facilitate an efficient charge separation between electron-rich and -poor bicontinuous networks.

The integrity and purity of **BTT(NDI)**_**3**_ and its precursors were confirmed by ^1^H NMR, ^13^C NMR, and matrix-assisted laser desorption/ionization time-of-flight mass spectrometry (MALDI-TOF-MS) (see the Supporting Information for the synthetic procedure and characterization).

### Spectroscopic and Structural Characterization

The self-assembly of **BTT(NDI)**_**3**_ was investigated in both toluene and THF solutions by absorption, circular dichroism (CD), and infrared spectroscopy.

**Figure 1:**
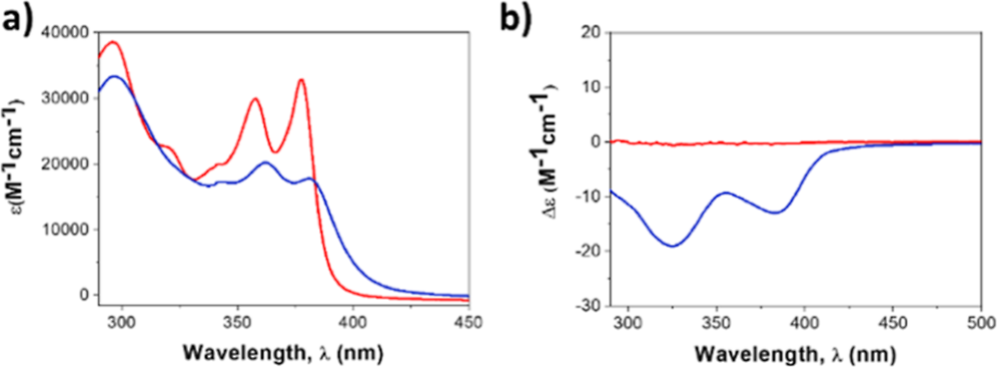
(a) Absorption and (b) CD spectra of **BTT(NDI)_3_** (1.0 × 10^–5^ M) recorded at 298 K in THF (red) and toluene (blue).

The absorption spectrum of NDI in **BTT(NDI)**_**3**_ in apolar solvents, such as toluene, shows less intense and slightly red-shifted absorptions at 361 and 381 nm relative to those at 357 and 377 nm in THF (Figure 1a). This strongly suggests stacking between electron-accepting NDIs in toluene. Absorptions related to the BTT core of **BTT(NDI)**_**3**_ were masked by the solvent cutoff at 284 nm. Bathochromic shifts as the ones observed indicates J-aggregating NDIs with a slipped arrangement between their aromatic cores. This contrasts similar NDI aggregates, in which a rigid benzene spaces NDIs from a benzene-1,3,5-tricarboxamide (BTA) central core.^47^

The **BTT(NDI)**_**3**_ self-assembly was also followed by CD spectroscopy. Solutions of **BTT(NDI)**_**3**_ in THF remained CD silent, while for those in toluene a pronounced Cotton effect was found in the CD spectra (Figure 1b). This supports the notion of excitonically coupled and helically ordered chromophores with a preferred helical conformation. Implicit is the transfer of chirality from the peripheral side chains to the chromophore (Figure 1b).^47^ In summary, our CD measurements suggest the lack of aggregation in THF, in line with the absorption measurements, and formation of chiral aggregates in toluene.

FT-IR spectra of **BTT(NDI)**_**3**_ in both the solid state and in toluene displayed a significant shift of the amide–NH stretching vibration, at 3261 cm^–1^ relative to 3317 cm^–1^ in THF. We conclude at this point the formation of 3-fold hydrogen bonded aggregates between amides, which enforces the cofacial arrangement of the BTT central cores (Figure S1, Supporting Information).^39,48^ Optimized (B3LYP/3-31G) structure of a tetrameric stack of **BTT(NDI)**_**3**_, where the peripheral alkoxy groups were removed, confirmed the structural arrangement of the supramolecular fibers (Figure S2, Supporting Information).

Our absorption, CD, and FT-IR measurements confirmed that **BTT(NDI)**_**3**_ self-assembles in toluene. Hereby, the benzotrithiophene tricarboxamides cores stack in a plane-to-plane fashion via head-to-tail hydrogen bonding and the amides adopt a helical conformation. The helical pitch forces the outer NDIs to stack in a laterally slipped fashion (typical of J-aggregates).

**Figure 2:**
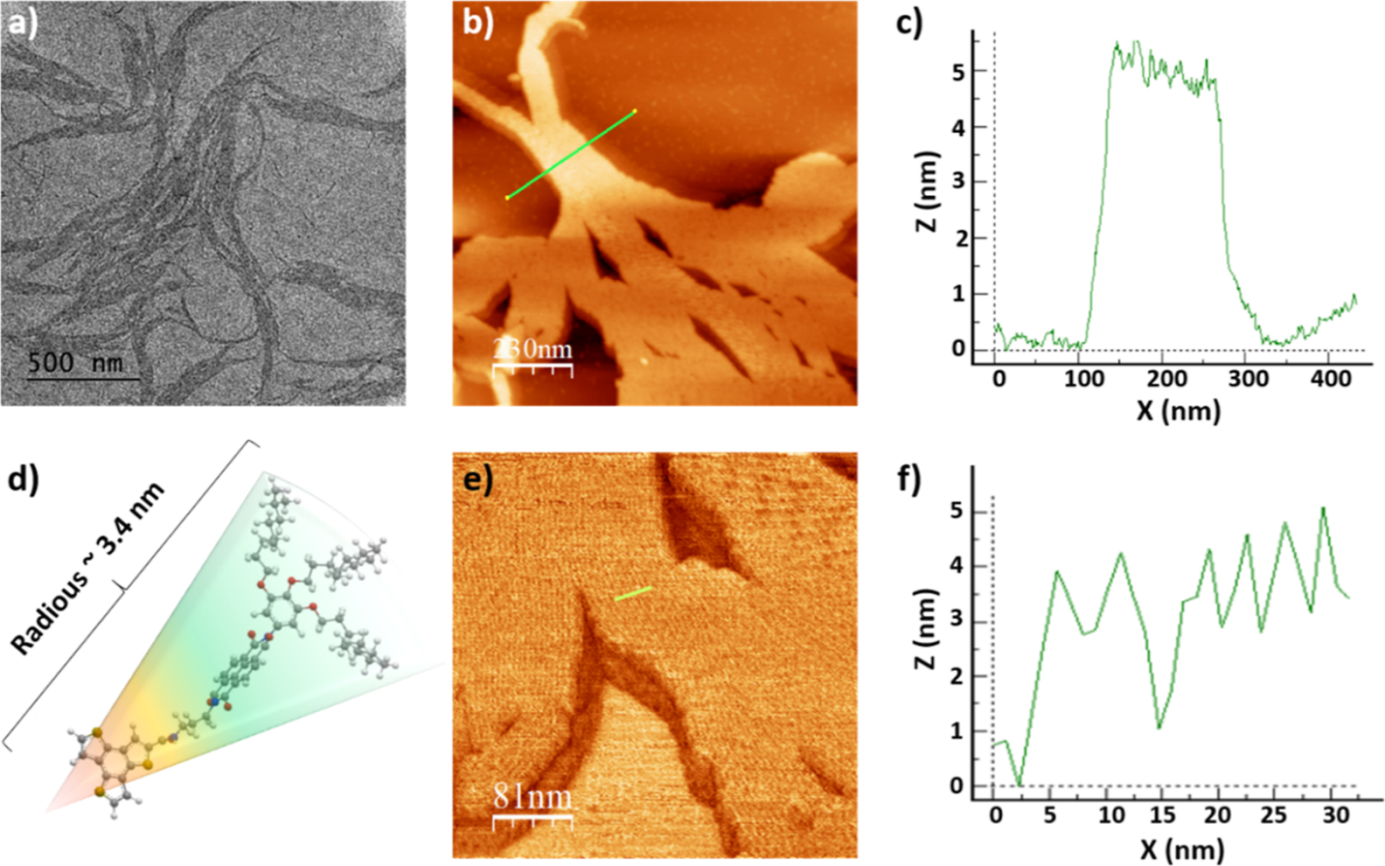
Characterization of self-assembled **BTT(NDI)_3_** nanofibers. (a) TEM image of a sample prepared from a 2.5 × 10^–6^ M drop-cast toluene solution. (b) AFM image (topographical scan) of **BTT(NDI)_3_** nanofiber bundles, prepared from a 1.5 × 10^–6^ M drop-cast toluene solution. (c) Height profile along the green line in (b). (d) Energy minimized structure of **BTT(NDI)_3_** in its extended form. Dark gray, carbon atoms; light gray, hydrogen atoms; red, oxygen atoms; blue, nitrogen atoms; yellow, sulfur atoms. (e) Zoom-in of (b). (f) Height profile along the green line in (e).

A closer inspection of the morphology of the **BTT(NDI)**_**3**_ aggregates by transmission electron microscopy (TEM) revealed the presence of fibrillar assemblies [Figures 2a and S3 (SI)]. The diameters of these fibers are typically multiples of 5 nm, which is in sound agreement with the molecular dimensions of the monomers. Their length was in the range of a few hundred nm to μm. A high tendency to form bundles of closely aligned fibers was observed. Atomic force microscopy (AFM) led to the same conclusions. Here, parallel fibers show a uniform height of 5 nm with a top-to-top peak distance of ∼5 nm [Figures 2b–f and S4 (SI)]. Further evidence of the elongated nature of the self-assembled architectures was obtained from small-angle X-ray scattering (SAXS) experiments carried out on 10^–4^ M toluene solutions of **BTT(NDI)**_**3**_ (Figure S5). The scattering data could fit the equation for a cylindrical form factor (i.e., a high aspect ratio morphology) with a radius of ca. 14 nm (Figure S5), in nice agreement with the conclusions previously drawn from TEM and AFM analyses. The higher concentration used in the SAXS experiments, which was necessary to increase the contrast, could result in an increased tendency to form bundles. This could explain the high value of the cylinder radius determined by SAXS.

All of the aforementioned confirms the formation of cylindrical 1-D supramolecular polymers, in which the alkyl chains of parallel fibers interdigitate.

### Thermodynamic Study of the Supramolecular Nanofibers

The function of supramolecular polymers relates closely to their thermodynamic stability and internal order, and, in turn, to the polymerization mechanism of the monomeric units.^49,50^ To study the thermodynamic aspects linked to the self-assembly of **BTT(NDI)**_**3**_ in toluene, we monitored first the spectroscopic features at variable temperatures. However, absorption and CD signals corresponding to the peripheral NDIs barely changed upon heating the solutions, even going beyond 370 K the aggregates are stable at low monomer concentrations (Figure S5, SI).

**Figure 3:**
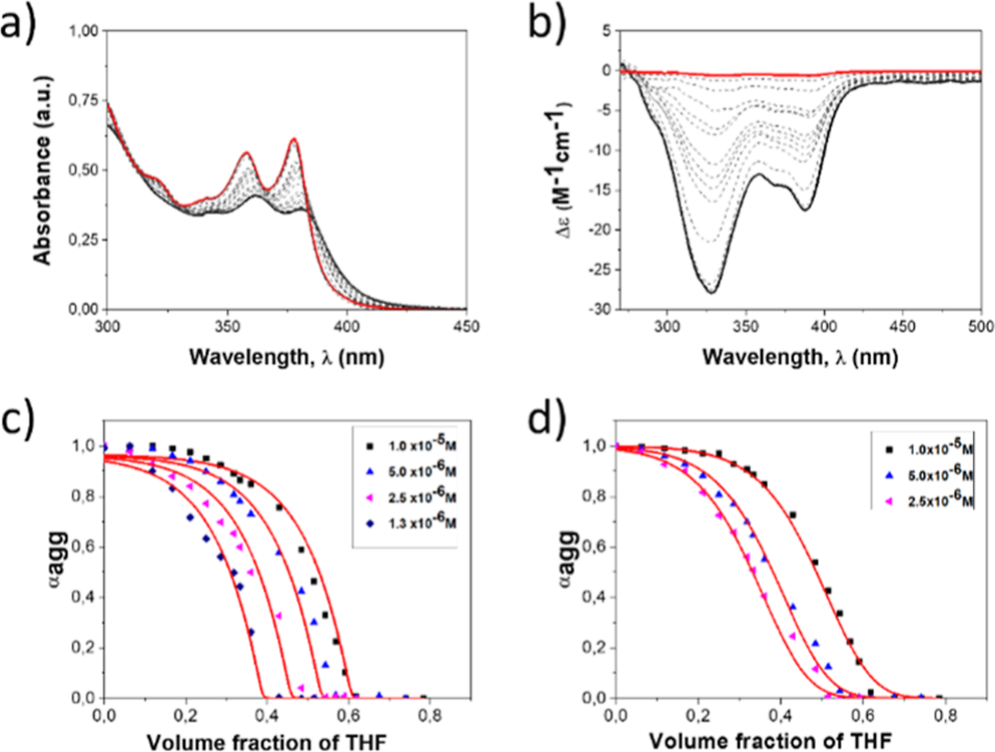
Titration of a toluene solution of **BTT(NDI)_3_** (blue, 1.0 × 10^–5^ M) until reaching the disaggregated state (red) by stepwise addition of THF and keeping the **BTT(NDI)_3_** constant recorded by (a) absorption and (b) CD spectroscopy at 298 K. Polymerization curves obtained after denaturation of the aggregates in toluene by increasing the molar fraction of THF to different concentrations determined by (c) absorption and (d) CD at 298 K. Spectroscopic measurements were carried out at 378 nm for the absorption spectra and 328 nm for the CD spectra. Polymerization curves were fitted with the SD model^50^ (red line).

To dissolve **BTT(NDI)**_**3**_, we turned to a solvent-denaturation method (SD).^51^ Throughout these experiments, the volume fraction of a “good” solvent such as THF was gradually increased to cause the disruption of the aggregates. As shown in Figure 3a,b, absorption as well as CD spectra drastically changed upon the stepwise addition of THF to toluene solutions of **BTT(NDI)**_**3**_. All together, the spectral changes support the transition from an aggregated form to a molecularly dissolved monomer as the spectroscopic features resemble those recorded in pure THF. Moreover, isosbestic points throughout the additions in absorption spectra point to an equilibrium between the aggregated and monomeric species (Figure 3a).

Nonsigmoidal curves were obtained by plotting the degree of aggregation (α_agg_) vs the volume fraction of THF (see Figure 3c,d). The resulting curves were fit to a model developed by de Greef, Meijer, and co-workers (Figure 3c,d and Tables 1 and S6) for the nucleation-elongation polymerization mechanism.^50^ From the fits, the Gibbs free energy gain upon monomer addition (Δ*G*′) was determined using the volume fraction (*f*) of the “good” solvent. Next to it, the ability of the “good” solvent to associate with the monomer as a means to destabilize the supra-molecular aggregated species (*m*), the equilibrium nucleation (*K*_n_), and the elongation (*K*_e_) constants, and the derived degree of cooperativity σ (*K*_n_/*K*_e_) were also obtained (Table 1). The application of the SD model revealed that **BTT(NDI)**_**3**_ forms supramolecular fibers in toluene with a 50 to 5-fold smaller nucleation constant (*K*_n_) with respect to the elongation step (*K*_e_). The differences between absorption and CD experiments are considerable, that is, on the order of one magnitude. A likely explanation is based on the fact that the experiments were performed using the absorption of the peripheral NDIs. Notably, chiral disposition of NDIs evolve slightly differently from J-aggregating.

**Table 1:** Thermodynamic Parameters Obtained From Global Fitting of the Temperature-dependent Absorption (λ_abs_ = 378 nm), and CD (λ = 328 nm) Data for **BTT(NDI)_3_** in Toluene at Different Concentrations on the Basis of the SD Model^51^

	Δ*G* [kJ mol^–1^]	*m*	σ^a^	Δ*G*′^a^ [kJ mol^–1^]	*K*_e_^a^ [M^–1^]	*K*_n_^a^ [M^–1^]
UV	–42.9	23.1	0.07	–38.3	5.2 × 10^6^	3.6 × 10^5^
CD	–45.7	25.7	0.5	–40.6	1.3 × 10^7^	6.5 × 10^6^

a Δ*G*′, *K*_e_, *K*_n_ and σ were calculated at 298 K for *f* = 0.2.

The cooperative polymerization mechanism observed for **BTT(NDI)_3_** is highly related to the formation of directional H-bonding providing highly ordered one-dimensional fibers.

### Characterization of the n/p-Material

**Figure 4:**
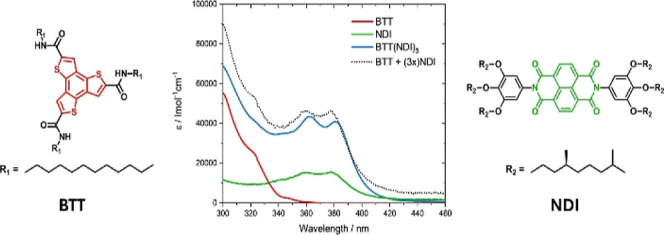
Absorption spectra of **BTT** (blue), **NDI** (red), **BTT(NDI)_3_** (green), and the superimposition of 1x BTT and 3x NDI (black) in toluene.

After the synthesis of **BTT(NDI)**_**3**_ its absorption spectrum and those of the individual **BTT** and **NDI** were carefully analyzed (Figure 4).

The dominant absorption of **BTT** features a maximum at 300 nm (ε_300 nm_ = 55,400 l mol^–1^ cm^–1^) and shoulders at 322 and 347 nm. Going beyond 370 nm, **BTT** lacks any appreciable absorption. **NDI** (Figure 4) shows maxima, albeit of moderate intensity, at 360 and 377 nm (ε_377 nm_ = 15,500 l mol^–1^ cm^–1^). These are, however, broader than those known for monomeric NDIs. **NDI** aggregation by π–π stacking is the likely cause, which is a known phenomenon especially in aromatic hydrocarbon solvents such as toluene.^52,53^ To compare the individual components with **BTT(NDI)**_**3**_, we added the absorption of one **BTT** and three **NDIs**. This assisted in figuring out possible ground-state interactions in **BTT(NDI)**_**3**_. In particular, the **BTT(NDI)**_**3**_ absorption is weaker and 4 nm red-shifted relative to the sum of **BTT** and **NDI**. This suggests moderate ground-state interactions between the individual components.

**Figure 5:**
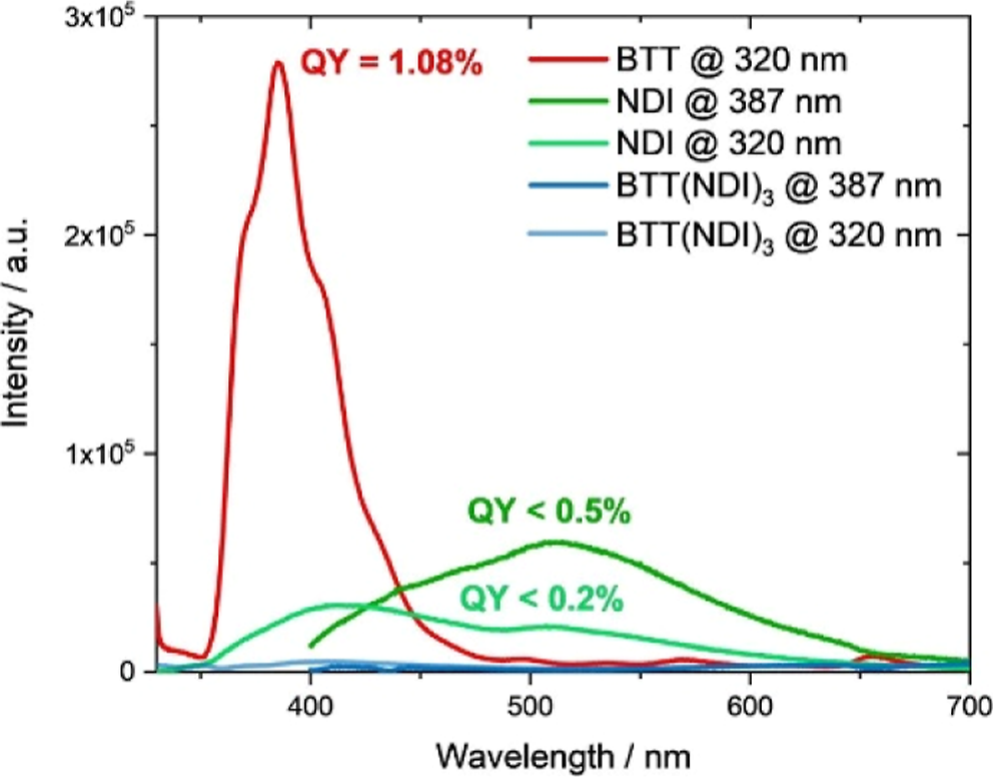
Fluorescence spectra of **BTT** (blue, 5 × 10^–6^ M), **NDI** (red and light red, 1 × 10^–5^ M) and **BTT(NDI)_3_** (green and light green, 5 × 10^–6^ M) in toluene upon photoexcitation at 320 or 387 nm. Intensities are adapted to properly show differences in QYs. Toluene signals such as Raman scattering were subtracted from all spectra. (QYs below 1% are near the limit of our integrating sphere and are given as upper estimates.)

As a complement, excited state interactions were investigated by means of fluorescence measurements. Figure 5 shows the fluorescence spectra of **BTT**, **NDI**, and **BTT(NDI)**_**3**_ upon 320 or 387 nm photoexcitation.

A fluorescence maximum for **BTT** is noted at 386 nm with minor shoulders between 400 and 500 nm. Its fluorescence quantum yield (QY) is 1.08%. The photoexcitation of **NDI** at 320 nm shows two fluorescent maxima located around 415 and 512 nm with a QY of <0.2%. Photoexcitation of **NDI** at 387 nm leads to the decrease and increase of the 415 and 512 nm maxima, respectively, accompanied by a rise of the QY to <0.5%. As the absorption assays suggest, NDI aggregation is active and the red-shifted fluorescence infers excimer-type interactions in the aggregates.^54,55^ To this end, the 415 nm maximum is assigned to a monomer-like fluorescence with an overall Stokes shift of 38 nm. Much larger is the Stokes shift, namely 135 nm, for the red-shifted 512 nm maximum due to the excimer fluorescence. In stark contrast, a quantitative quenching of the fluorescence is observed for **BTT(NDI)**_**3**_ at both photoexcitation wavelengths. In other words, new deactivation pathways are activated upon photoexciting in **BTT(NDI)**_**3**_ that are absent in **BTT** and **NDI**.

Furthermore, to determine the values of the reduction and oxidation potentials of each one of the components separately, a cyclic voltammetry (CV) study was performed (see Figures S6–S9). The energetic values of the corresponding HOMO and LUMO levels clearly showed the possibility that intramolecular charge transfer phenomena occurred between both chromophores (**BTT** and **NDI**), although, as in UV experiments certain degree of aggregation could be observed **in BTT(NDI)**_**3**_ cyclic voltammogram (see Figures S8–S10).

### Transient Absorption Spectroscopy

**Figure 6:**
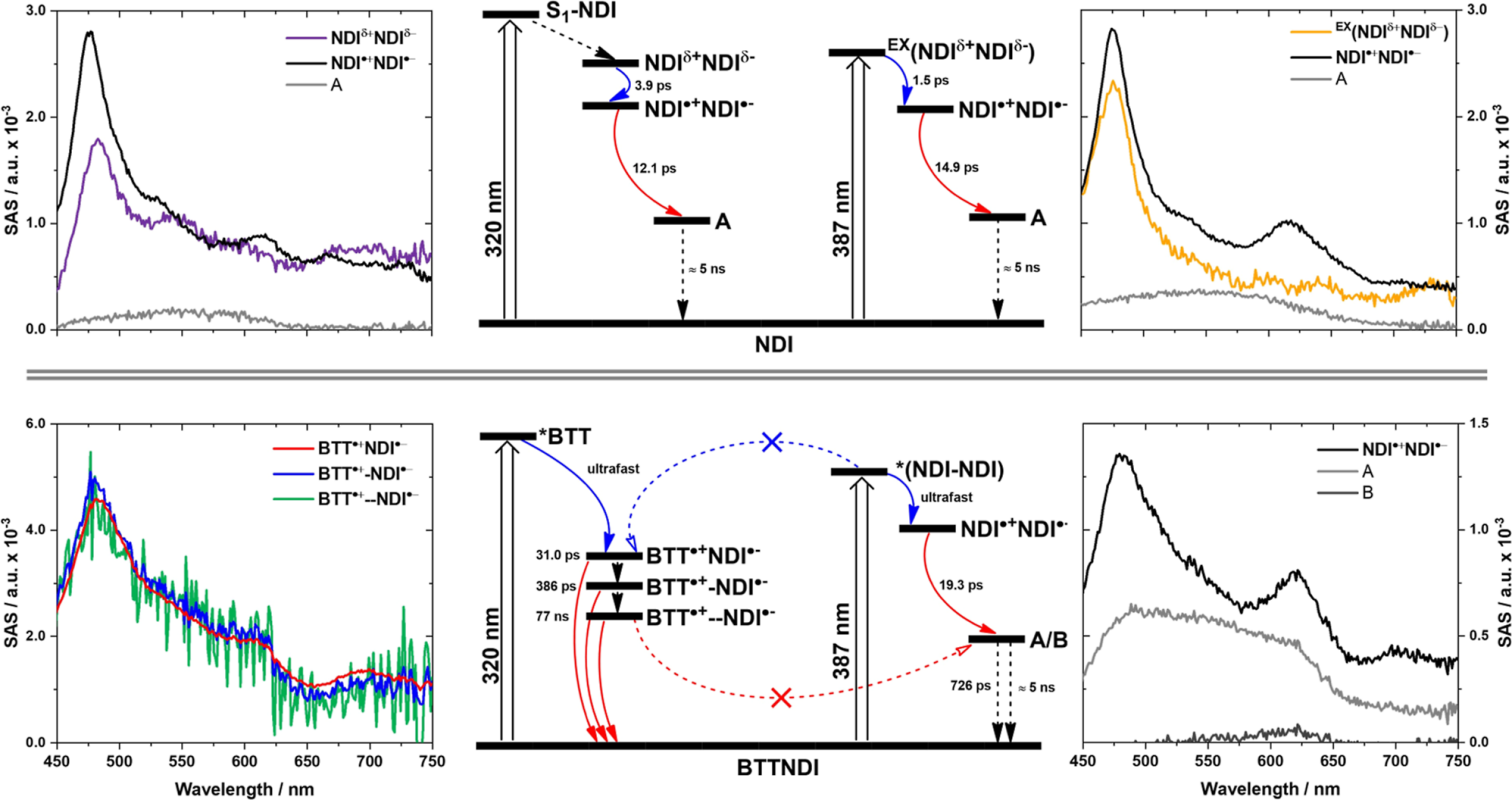
Left and right side: SAS obtained upon chirp corrected GloTarAn analysis of the raw data of **NDI** excited at 320 nm (top left), **NDI** excited at 387 nm (top right), **BTT(NDI)**_**3**_ excited at 320 nm (bottom left), and **BTT(NDI)_3_** excited at 387 nm (bottom right) with NDI^δ+^NDI^δ−^ (purple), ^EX^(NDI^δ+^NDI^δ−^) (yellow) NDI^•+^NDI^•–^ (black), BTT^•+^NDI^•–^ (red), BTT^•+^-NDI^·–^ (blue), BTT^•+^--NDI^•–^ (green), product A (light gray), and product B (dark gray). Center: Derived Jablonski diagrams for **NDI** (top) and **BTT(NDI)_3_** (bottom) upon photoexcitation at 320 or 387 nm. Charge separation and charge recombination pathways are highlighted in blue and red, respectively.

To gain further insights into the excited state dynamics upon photoexcitation of **BTT(NDI)**_**3**_, transient absorption spectroscopy (TAS) was conducted. A photoexcitation wavelength of 320 nm was chosen to photoexcite both **BTT** and **NDI**, whereas photoexcitation at 387 nm selectively photoexcites **NDI**. As seen in Figure 5, selective photoexcitation of **BTT** is impossible. Notably, photoexcitation at 320 nm also excited the solvent, i.e. toluene. This required a parallel pathway in the kinetic model for the GloTarAn^56^ fitting, which was used to analyze the TAS spectra.^57^ Jablonski diagrams for the photoexcitation of **NDI** and **BTT(NDI)**_**3**_, which were constructed based on our spectroscopic results, and the corresponding SAS spectra are shown in Figure 6.

Starting with the photoexcitation of **BTT** at 320 nm, overall weak transients emerge (Figures 6 and S11) at any given time delay to toluene-centered excited states. Best fits of the raw data yielded two **BTT**-centered species in parallel to one toluene-centered species. The latter is characterized by a broad excited state absorption (ESA) that maximizes at 465 nm and that is with 0.8 ps short-lived. For the earlier two, we noted ESA maxima at 560 and 470 nm and lifetimes of 1.49 and 556 ns, respectively. We assign them to higher-lying excited (**BTT) and lower-lying excited (*BTT) states.

Photoexcitation of **NDI** at 320 nm gives rise to a kinetic model for three different species (Figures 6 and S12). Initially, a transient with ESA maxima at 483, 546, and 690 nm is formed as first species. It is attributed to a symmetry broken charge transfer (CT) state, that is, (NDI^δ+^NDI^δ−^), rather than the singlet excited state (S_1_) of NDI. Our assignment is supported by the notion that (CT) has little resemblance with (S_1_) or any triplet excited state (T_1_) features of NDIs.^58,59^ In fact, its features are much more similar to the state that is formed next, namely the symmetry broken charge separated state (NDI^•+^NDI^•–^). This implies that the (S_1_) lifetime must be shorter than the temporal resolution of our instrumentation. Charge separation to afford (NDI^•+^NDI^•–^) takes about 3.5 ps. Hereby, NDI^•–^ is identified by its 475, 530, 615, 665, and 730 nm ESA fingerprints, which are in sound agreement with literature.^59,60^ Spectral features of NDI^•+^ have been reported to be nearly identical to those of NDI^•–^.^59^ The only notable difference is the lack of the 665 nm feature. (NDI^•+^NDI^•–^) decays with 13.1 ps via charge recombination to yield the third species. We label this as A. A has a lifetime of roughly 5 ns and a broad ESA across the visible region. Charge recombination under the simultaneous recovery of the ground state should be the most plausible deactivation pathway for (NDI^•+^NDI^•–^). But, considering the lack of ground-state bleaching (GSB), we rule out a direct ground-state recovery—at least as a major pathway.

Photoexcitation of **NDI** at 387 nm and fitting the raw data with a kinetic model gives rise to three species, which are comparable to those seen in the 320 nm photoexcitation experiments (Figures 6 and S14). The species preceding (NDI^•+^NDI^•–^) differs slightly from (NDI^δ+^NDI^δ−^) found in the 320 nm photoexcitation experiments. It is attributed to an excimer-type species of **NDI** [^EX^(NDI^δ+^NDI^δ−^)], which was observed in the fluorescence experiments—vide supra—and is based on a partial delocalization of charges. ^EX^(NDI^δ+^NDI^δ−^) as first species. It undergoes full charge separation within 1.5 ps. (NDI^•+^NDI^•–^) as second species, which then recombines with 14.9 ps and transforms to A, which was observed earlier, as third species.

When turning to photoexcitation of **BTT(NDI)**_**3**_ at either 320 or 387 nm, important differences are noted when comparing these two different excitation wavelengths. Photoexcitation at 387 nm (Figures 6 and S15), that is, the selective photoexcitation of **NDI**, directly populates the charge separated state (NDI^•+^NDI^•–^) that was seen for **NDI** as second species. Spectroscopic evidence are ESA maxima at 481, 620, and 700 nm. Its lifetime is 19.3 ps. In **BTT(NDI)**_**3**_, its helical arrangement facilitates edge-to-edge rather than face-to-face interactions and provides the necessary electronic coupling for charge separation. As such, (NDI^•+^NDI^•–^) evolves from (S_1_) of **NDI**, which is, however, outside of the temporal resolution of our instrumentation. By means of charge recombination, the second and third species are populated in parallel. Both are characterized by rather broad ESAs, which range from 450 to 650 nm for A, and, which maximize at 620 nm for B. Their lifetimes are 726 ps and >5 ns. These are assigned to charge recombination products of **NDI** as seen in experiments with **NDI** and are labeled as A and B.

Upon 320 nm photoexcitation of **BTT** in **BTT(NDI)**_**3**_ we used a kinetic model based on three species to fit the raw data. Importantly, all three species (Figures 6, S16 and S17) revealed identical ESAs, namely maxima at 485, 540, 610, and 695 nm. At first glance they resemble those seen for (NDI^•+^NDI^•–^) upon either 387 nm photoexcitation of **BTT(NDI)**_**3**_ or 320 nm photoexcitation of **NDI**. However, the fact that we note three different lifetimes makes us believe that the charge-separated state (BTT^•+^-NDI^•–^) is formed. Spectroelectrochemical oxidation experiments with **BTT** failed to yield characteristic fingerprints. A weakly absorbing BTT^•+^ is the only rationale. Independent support for the notion of (BTT^•+^-NDI^•–^) rather than (NDI^•+^NDI^•–^) comes from the important observation that in the 320 nm photoexcitation experiments the charge recombination products A and B that were seen for (NDI^•+^NDI^•–^) are absent. In other words, it is imperative to photoexcite **BTT** and not **NDI** to power charge separation between **BTT** and **NDI**. An increase of the longest (BTT^•+^NDI^•–^) lifetime by up to 3 orders of magnitude when compared to (NDI^•+^NDI^•–^) in 387 nm photoexcitation experiments with **BTT(NDI)**_**3**_ and a multiphasic decay suggests a charge delocalization along the π–π stacks of **BTT(NDI)**_**3**_. Holes are likely to be transferred from one **BTT** to another, while electrons are transported along the **NDI**s, which slows down charge recombination.

## Conclusions

A newly C_3_-symmetric disc shaped chromophore comprising electron accepting naphthalene diimides anchored on an electron donor **BTT** core (**BTT(NDI)**_**3**_) is designed and synthesized. Monomers of **BTT(NDI)**_**3**_ self-assemble in apolar solvents into highly ordered, chiral supramolecular fibers in a cooperative fashion. Hereby, the combination of π–π and 3-fold hydrogen-bonding interactions forces the covalently attached Ds and As to homostack and to generate supramolecular structures. In the latter, **BTT**s stack in a plane-to-plane fashion, while the outer **NDI**s are forced to form J-aggregates due to the helical nature of the fibers. Overall, the unique internal order within the double-cable supramolecular structures is the basis for high charge mobilities. As a matter of fact, transient absorption experiments demonstrate that an electron transfer evolves from the **BTT**s to the **NDI**s upon photoexcitation. Hereby, the (BTT^•+^-NDI^•–^) lifetimes increase by up to 3 orders of magnitude when compared to those of (NDI^•+^NDI^•-^) in **BTT(NDI)**_**3**_ upon 320 nm photoexcitation. Of great value is the fact that multiphasic decay of (BTT^•+^-NDI^•–^) is detected. This speaks for ambipolar pathways for charge carriers that facilitate the delocalization of holes and electrons along the respective **BTT** and **NDI** stacks.

Overall, our supramolecular approach paves the way for the preparation of versatile coaxially nanosegregated functional supramolecular polymers. They give rise to continuous pathways for the free electrons and holes to travel to their respective electrodes. As a consequence, losses due to charge recombination in the active layer of organic photovoltaic devices should be minimized.

## Supporting Information Available

All of the synthetic details and molecular characterization as well as the device manufacturing and the details of the ferroelectric measurements (PDF)
