# Supplementary material for: Long-Lived Charge Carrier Photogeneration in a Cooperative Supramolecular Double-Cable Polymer
Source: J Am Chem Soc. 2024 Oct 22;146(44):30272–80. doi: 10.1021/jacs.4c09637 (PMC11544610; doi:10.1021/jacs.4c09637)
Supplement: Supplementary file 1 — ja4c09637_si_001.pdf [file ja4c09637_si_001.pdf]

Supporting Online Material for

Long-Lived Charge Carrier Photogeneration in a Cooperative Supramolecular Double-Cable Polymer.

Jan Joseph,‡ José Augusto Berrocal,§ Nicolás M. Casellas,† Dirk M. Guldi, *, ‡ Tomás Torres, *, †, ∞, ^ Miguel García-Iglesias *, †, ~

‡ Friedrich-Alexander-Universität Erlangen-Nürnberg FAU Profile Center Solar Department of Chemistry and Pharmacy Interdisciplinary Center for Molecular Materials (ICMM) Egerlandstr. 3

91058 Erlangen, Germany. E-mail: dirk.guldi@fau.de

§ Institute of Chemical Research of Catalonia (ICIQ), Barcelona Institute of Science and Technology (BIST), Av.da Països Catalans 16, 43007 Tarragona, Spain.

~ QUIPRE Department, Nanomedicine-IDIVAL, Universidad de Cantabria, Avd. de Los Castros, 46, 39005 Santander, Spain. giglesiasm@unican.es

† Departamento de Química Orgánica, Universidad Autónoma de Madrid, Cantoblanco, 28049 Madrid, Spain. E-mail: tomas.torres@uam.es

∞ Institute for Advanced Research in Chemical Sciences (IAdChem), Universidad Autonoma de Madrid (UAM), 28049 Madrid, Spain

^ IMDEA-Nanociencia, c/Faraday, 9, Cantoblanco, 28049 Madrid, Spain

Contents

Experimental details S2

1. Materials S2
2. Methods S2
3. Synthetic procedures S4

Supporting data ....

Figures S1 and S2 .... S16

Figures S3 and S4 .... S17

Figures S5 and S6 .... S18

Figures S7, S8 and S9 .... S19

Fig. S10 .... S20

Fig. S11 .... S21

Fig. S12 .... S22

Fig. S13 .... S23

Fig. S14 .... S24

Fig. S15 .... S25

Fig. S16 .... S26

Fig. S17 .... S27

Fig. S18 .... S28

References S29

Experimental details

1. Materials

Chemicals were purchased from commercial suppliers (SIGMA Aldrich and Alfa Aesar) and used without further purification unless stated otherwise. All solvents were of AR quality and purchased from either Scharlab or Carlo Erba. Dry THF was degassed and obtained after passing through an activated alumina column in a solvent purification system. Water was purified using an EMD Milipore Mili-Q integral water purification system. Column chromatography was carried out on silica gel (Merk, kieselgel 60, 230-400 mesh, 60 Å). Reactions were followed by thin-layer chromatography on aluminium sheets precoated 0.25 mm, 60-F254 silica gel plates from Merck. All reactions were performed under an atmosphere of dry argon unless stated otherwise.

Column chromatography was performed using a Grace Reveleris instrument equipped with a UV-Vis and evaporative light scattering detectors.

2. Methods

ESI-HRMS spectra were obtained from an Applied Biosystems QSTAR equipment, PCI-HRMS on a Bruker MAXIS II spectrometer, and **MALDI-TOF HRMS** on a Bruker Reflex III spectrometer. **NMR** spectra were recorded with a BRUKER AVANCE-II (300 MHz) instrument and BRUKER DRX 500 MHz. The temperature was actively controlled at 298 K. Chemical shifts are measured in ppm using the signals of the deuterated solvent as the internal standard [CDCl3 calibrated at 7.26 ppm (1H) and 77.0 ppm (13C), DMSO-D6 calibrated at 2.50 ppm (1H) and 39.5 ppm (13C) and THF-D8 calibrated at 3.58 (1H)]. **Column chromatography** was carried out on silica gel Merck-60 (230-400 mesh, 60 Å), and TLC on aluminium sheets precoated with silica gel 60 F254 (Merck). **CD and UV-Visible** spectra were recorded with a JASCO V-815 equipment (measurement Information: data interval = 1 nm, data pitch = 1 nm, sensitivity = standard, D.I.T. = 1 sec, slit width = 1000 um). **Emission** spectra were recorded in a JASCO Fp-8600 equipment. Quartz cuvettes (1 and 0.1 cm path length) were used for the measurements. In these three instruments, the temperature was controlled using a JASCO Peltier thermostatted cell holder. Spectroscopic measurements were performed using quartz cuvettes (1 cm). Solutions were prepared by weighting the necessary amount of compound for a given concentration.

Transmission electron microscopy (TEM) measurements were performed on a JEOL JEM1010 electron microscope operating at an acceleration voltage of 100 kV. For the observation of aggregates, a drop of sample suspension was placed on formvar copper grids coated with carbon.

AFM images were taken from drop casted solutions in toluene onto HOPG employing a JPK Nanowizard II system working on dynamic mode.

Small angle X-ray scattering (SAXS) experiments were performed at the NCD-SWEET beamline of the ALBA synchrotron using X-ray photons with a wavelength of 1 Å and a flux of $1·10^{12}$ ph/s @ 250 mA. SAXS and WAXS scattering patterns were simultaneously collected using a Dectris Pilatus 1M 3s and Rayonix LX 255 HS respectively. SAXS sample-to-detector distance of 2.16 m were used giving an observed q range of 0.07 $nm^{-1} \leq q \leq 6.8\ nm^{-1}$. The solutions ($10^{-4}$ M) were measured in 2 mm borosilicate capillaries. The python pyFAI library was used to azimuthally average the resulting 2D images to obtain the intensity I(q) vs. q profiles. Standard data reduction procedures, i.e. subtraction of the solvent's contribution, were performed using the same software.

Experimental data were fit to a cylindrical form factor using the SasView 5.0.6 software package. The scale and background were set at 2.1875 * 10^{-7} nm-1 and 8.6923 * 10^{-8} nm^{-1}, respectively. The radius of the cylinder was the only optimized parameter (optimized value = 14.288 nm). Polydispersity was set at 0.2 in the fitting process.

Cyclic Voltammetry (CV) and Square Wave Voltammetry (SWV): Electrochemical measurements were performed on an Autolab PGStat 30 equipment using a three electrode configuration system. The measurements were carried out using freshly distilled THF solution containing 0.1 M tetrabutylammonium hexafluorophosphate (TBAPF6) and a concentration of 10^{-4} M of the corresponding compound. A glassy carbon electrode (3 mm diameter) was used as the working electrode, and a platinum wire and an Ag/AgNO3 (in CH3CN) electrode were employed as the counter and the reference electrodes, respectively. Ferrocene (Fc) was used as an internal reference and all the potentials were given relative to the Fc/Fc+ couple. Scan rate was 100 mV s1 unless otherwise specified.

Ultrafast transient absorption experiments were conducted using an Astrella-F-1K amplified Ti:sapphire femtosecond laser system from Coherent, operating at a repetition rate 1kHz, 5.5 W power (5 mJ pulse energy), pulse duration of 80 fs, with TA pump / probe Helios and EOS detection systems from Ultrafast Systems. White light was generated focusing a fraction of the fundamental 800 nm output onto a 2 mm sapphire crystal (Helios). A 1.2 mJ fraction of the fundamental is used for pump beam generation by a TOPAS Prime from Light Conversion with standard NirUVis extension. The pump energy was varied between 150 and 1000 nJ. The laser spot dimaeter was estimated to be 0.5 cm. Therefore, assuming a spherical shape, the area was ca. 0.196 cm^2. A depolarizer was placed in the pump beam to avoid rotational dynamics. Bandpass filters with ± 5 or ± 10 nm were used to ensure low spectral width and to exclude 800

nm photons. All measurements were conducted in a 2 mm quartz cuvette under argon atmosphere, using solutions with absorbances of 0.5-0.7 under continuous stirring. To analyze transient absorption data, we start with SVD and global analysis, using an all-sequential decay model that provides evolution associated spectra of potentially intervening species, to determine the number of decaying species that participate in the decay cascade. However, this does not necessarily yield differential spectra with genuine physicochemical meaning. Afterwards, a target analysis is applied, using specific target models that result in species associated difference spectra (SADS) with true physicochemical meaning. Obtained data were treated by SVD, global and target analyses using the R- package TIMP and GloTarAn.

3. Synthetic procedures

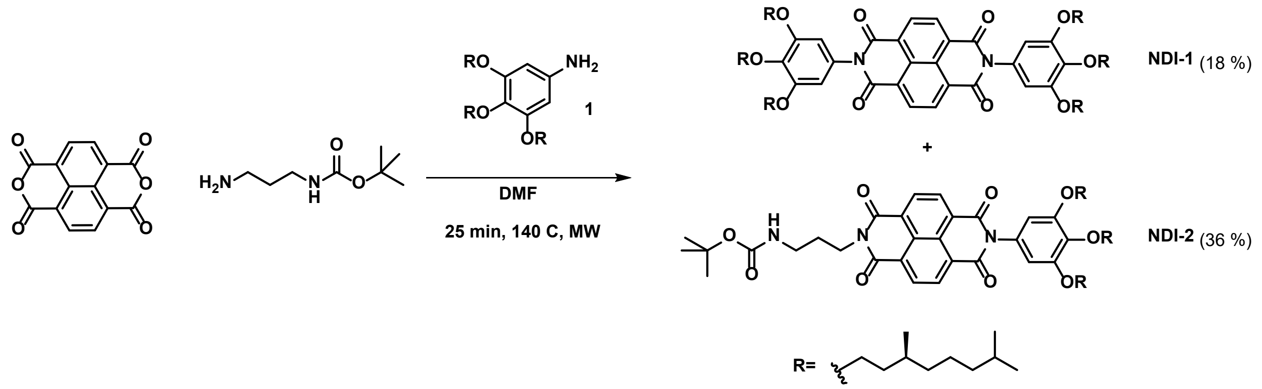

Scheme S1: Synthetic route for compounds **NDI-1 and NDI-2**.

Compounds NDI-1 and NDI-2

1,4,5,8-naphthalenetracarboxylic dianhydride (0.286 g, 1.07 mmol), aniline **1**[2] (0.60 g, 1.07 mmol) and N-Boc-1,3-propanediamine (0.186 g, 1.07 mmol) were placed in a 20 mL microwave vial equipped with a magnetic stirrer. Then, 7mL of dry DMF were added and the resulting mixture was firstly heated at 70 °C for 5 minutes and later the temperature is increased to 140 °C for 25 minutes in the microwave. After the reaction was completed, the solvent in the organic layer was removed under vacuum and the residue was purified by column chromatography on a silica gel column using a DCM/EtOAc (90/10 v/v) mixture as eluent affording two fractions of orange-red solids as **NDI-1**(0.261 g, 0.19, 18%) and **NDI-2** (0.367 g, 0.38 mmol, 36%).

Compounds NDI-1.

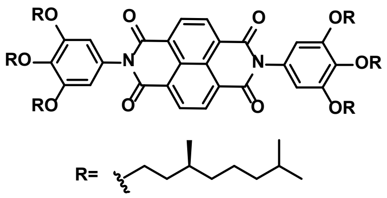

^{1}H-NMR (400 MHz, $CDCl_3$) δ: 8.80 (s, 4H), 6.47 (s, 4H), 4.09-3.93 (m, 16H), 2.0-1.5 (m, 40H), 2.0-1.5 (m, 63H). 0.96-0.84 (m, 60H). **^{13}C-NMR** (101 MHz, $CDCl_3$) δ: 163.11, 153.95, 138.76, 131.54, 129.53, 127.21, 106.80, 67.59, 39.43, 37.51, 36.56, 30.01, 28.15, 24.88, 22.76, 19.73. **FT-IR** (ATR) ν (cm^{-1}):3347, 2953, 2925, 2869, 1716, 1673, 1601, 1582, 1504, 1463, 1440, 1384, 1366, 1314, 1251, 1239, 1213, 1197, 1114, 1064, 978, 916, 881. **MS** (MALDI-TOF, DCTB) (m/z): [M•]$^-$ calcd $C_{86}H_{134}N_2O_{10}$: 1355.06; found 1355.05.

^{1}H NMR spectrum of NDI-1 in $CDCl_3$

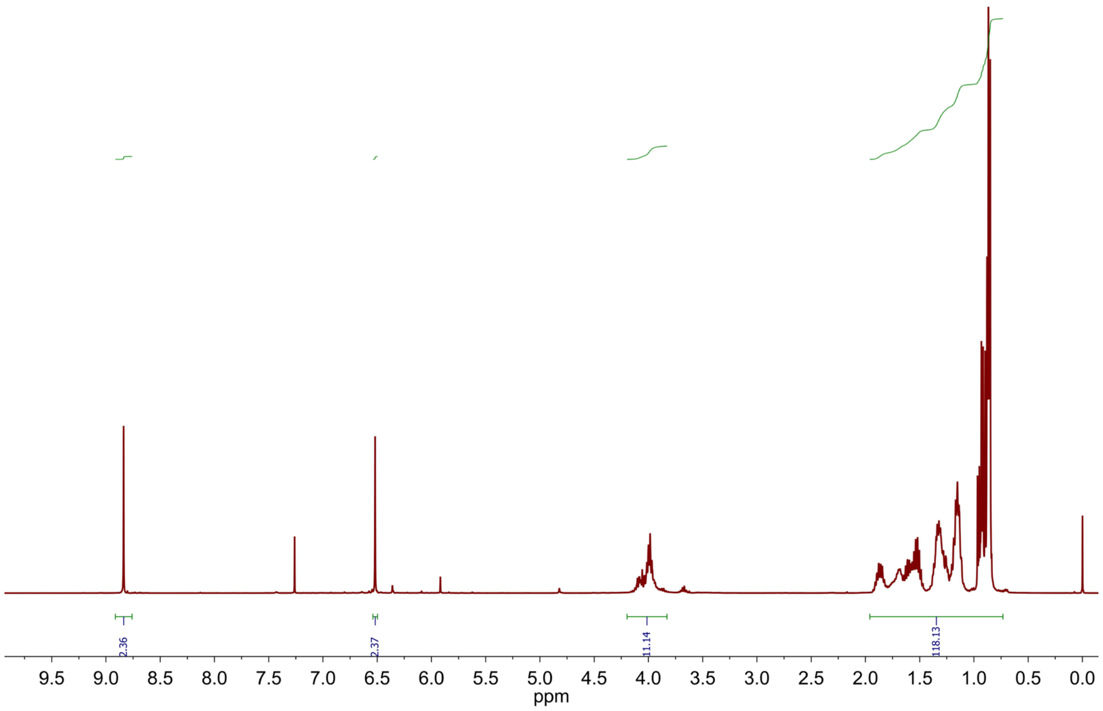

^{13}C NMR spectrum of NDI-1 in $CDCl_3$

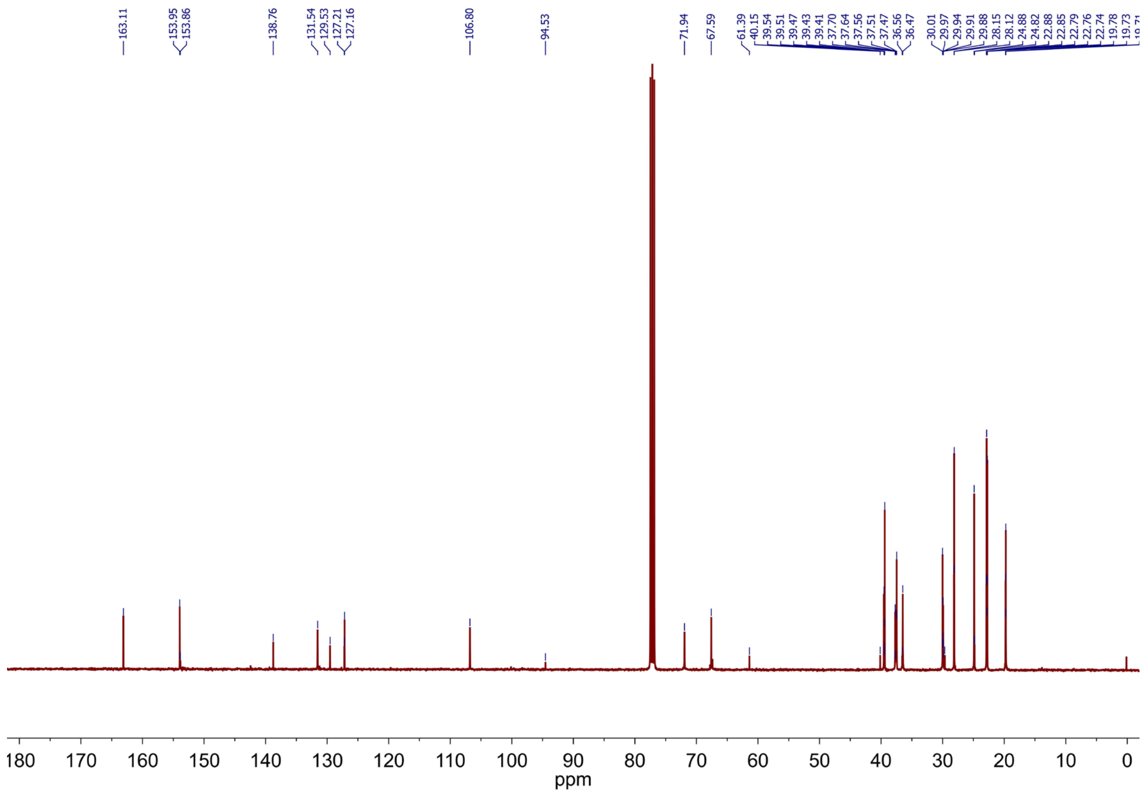

IR-FT spectrum of NDI-1 (solid state).

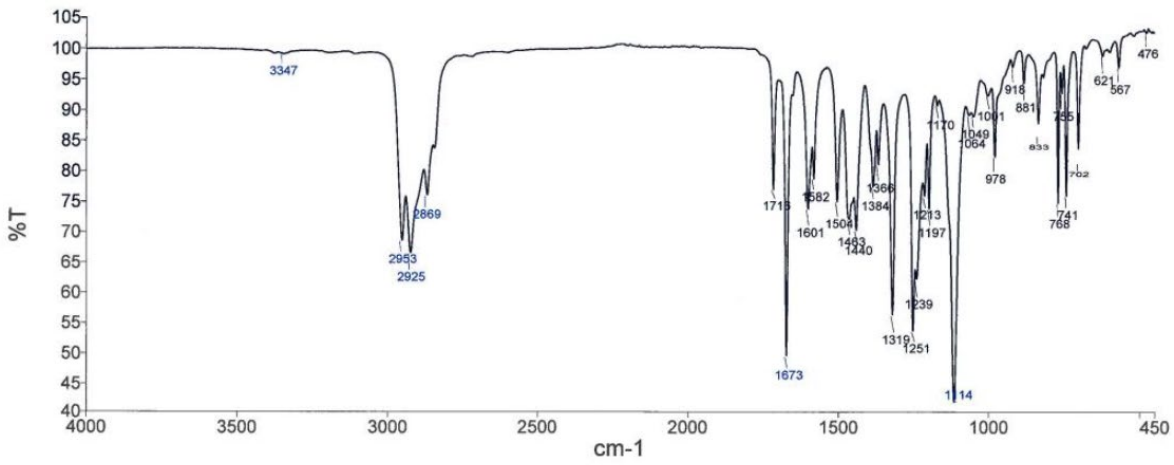

MALDI-TOF mass spectrum and isotopic pattern of NDI-1 using two different matrixes (CHCA and DCTB) .

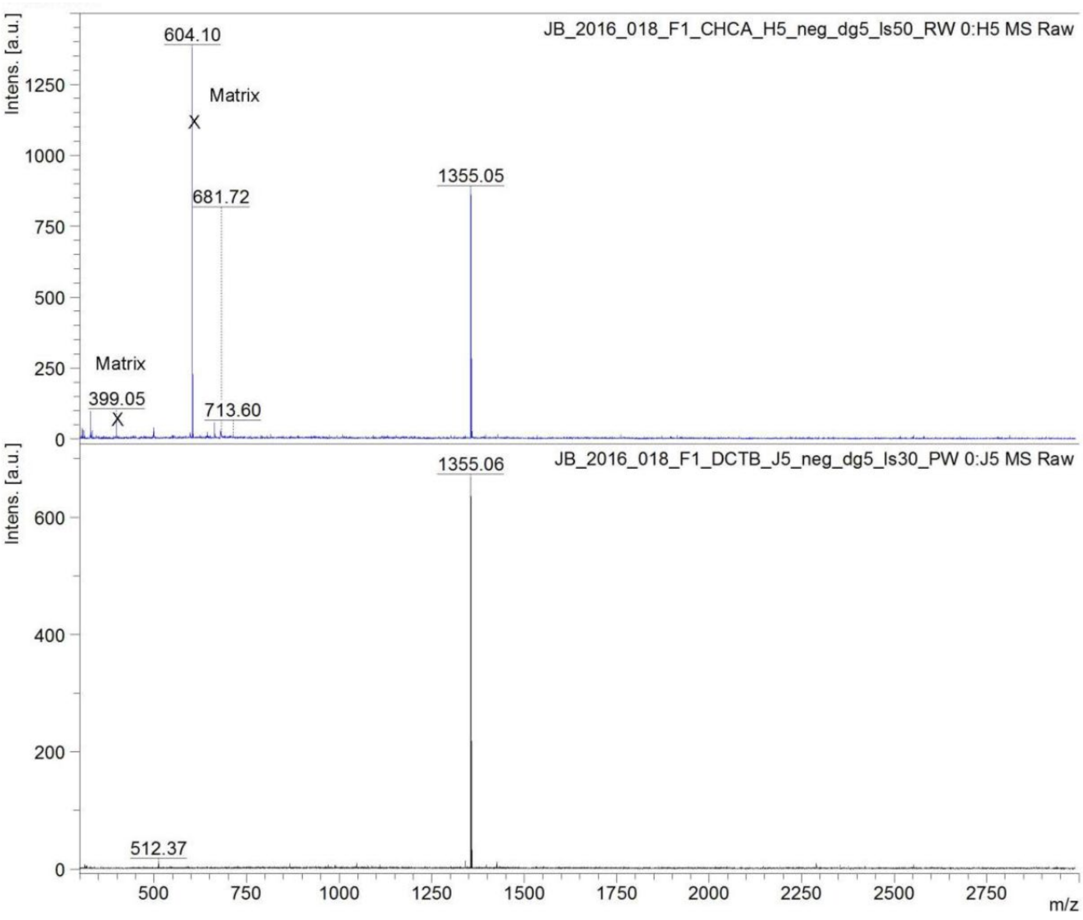

JB_2016_018_F1_CHCA_H5_neg_dg5_ls50_RW 0:H5 MS Raw
Intens. [a.u.]
604.10
Matrix
1355.05
681.72
Matrix
399.05
713.60
JB_2016_018_F1_DCTB_J5_neg_dg5_ls30_PW 0:J5 MS Raw
1355.06
512.37
m/z

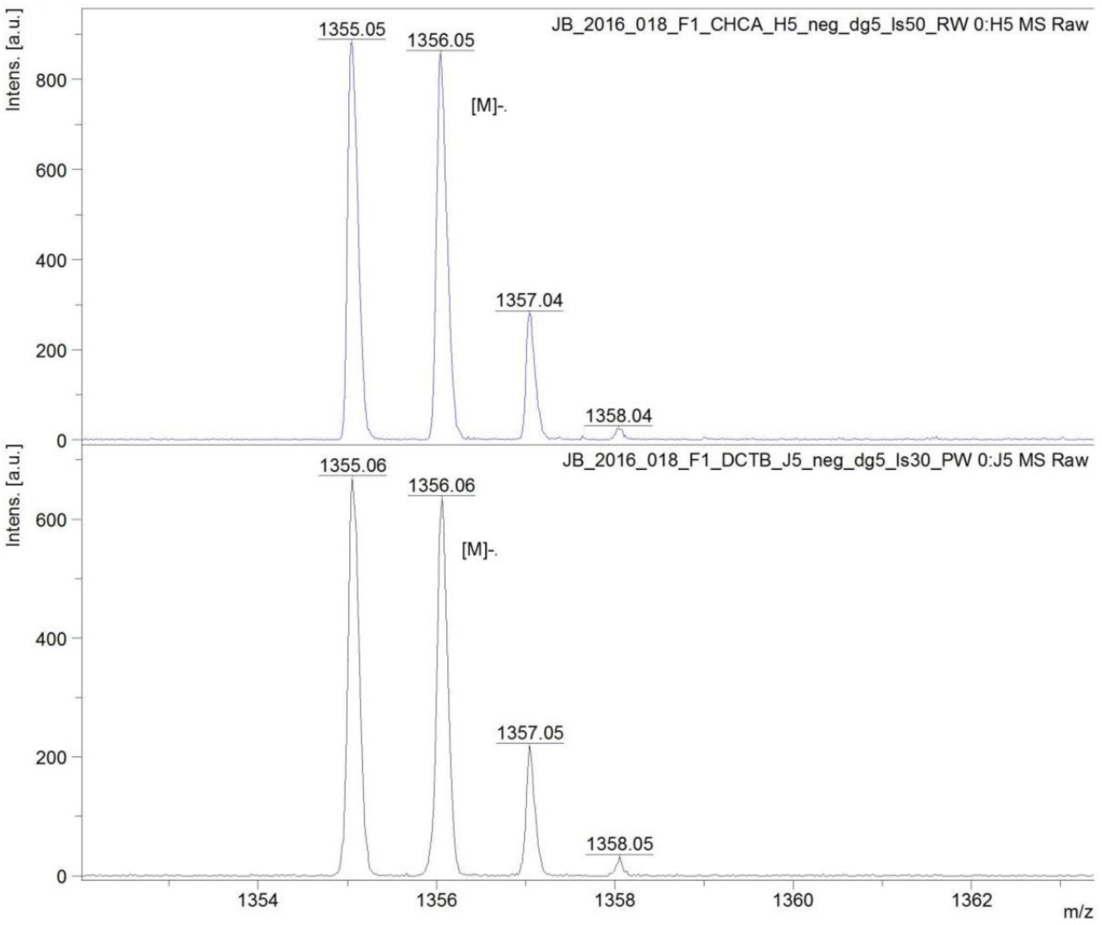

Compounds NDI-2.

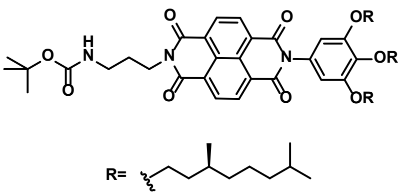

^{1}H-NMR (400 MHz, $CDCl_3$) δ: 8.78 (s, 4H), 6.50 (s, 2H) 5.16-5.01 (m, 1H), 4.30 (t, *J* = 6.6 Hz, 2H), 4.15 – 3.90 (m, 6H), 3.93-3.90 (m, 4H), 3.21 (q, *J* = 6.3 Hz, 2H), 1.98 (m, 2H) 1.83-1.81 (m, 6H,), 1.46 (s, 9H,), 1.29-1.25 (m, 20 H,) 0.96-0.84 (m, 30H). **^{13}C-RMN** (101 MHz, CDCl3, δ) 163.18, 163.10, 153.93, 138.73, 131.48, 131.34, 127.012, 127.05, 126.96, 106.79, 79.40, 71.93, 67.58, 39.53, 39.40, 38.47, 37.47, 36.47 30.01, 29.94, 29.91, 28.70, 28.58, 28.15, 28.12, 24.87, 22.88, 22.84, 22.75, 19.78. **FT-IR** (ATR) ν (cm^{-1}): 3385, 3083, 2953, 2926, 2869, 1708, 1664, 1600, 1580, 1503, 1452, 1438, 1384, 1366, 1334,1316, 1246, 1192, 1170,1113, 1064, 996, 976, 878, 766, 626, 572. **MS** (MALDI-TOF, DCTB) (m/z): $[M^{\bullet}]^-$ calcd $C_{58}H_{85}N_3O_9$: 967.61; found 967.64.

^{1}H NMR spectrum of NDI-2 in $CDCl_3$

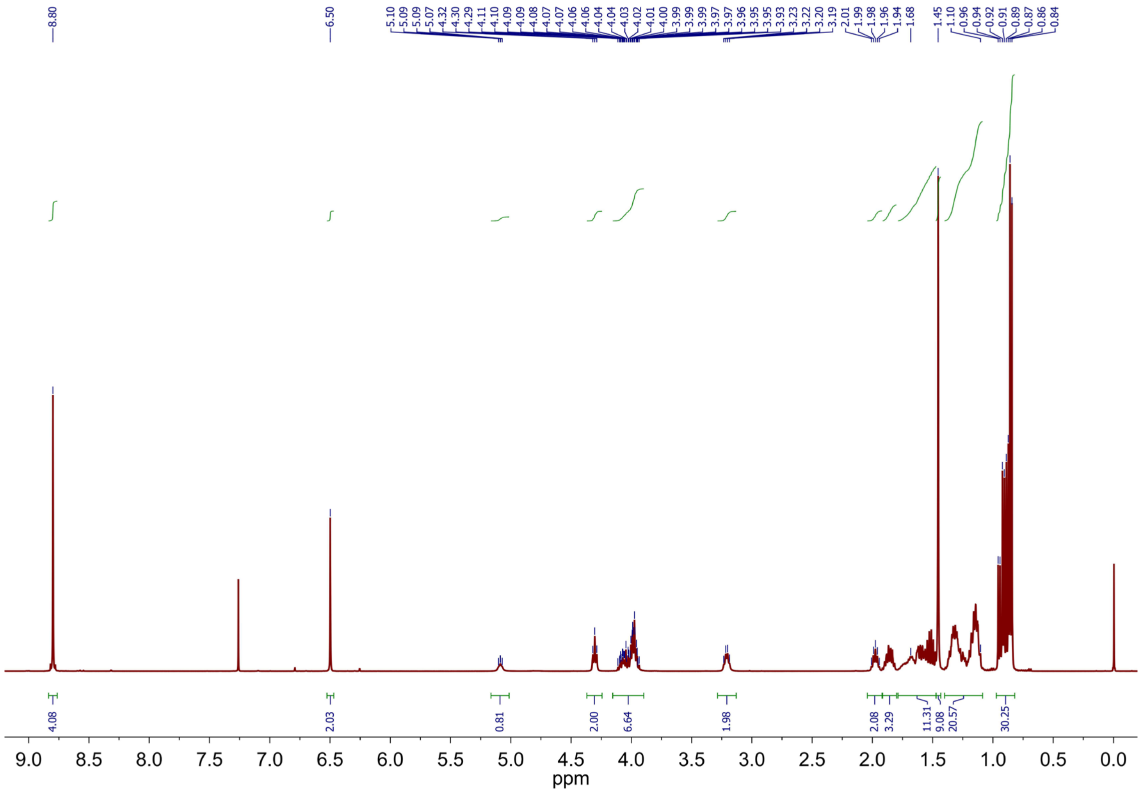

^{13}C NMR spectrum of NDI-2 in $CDCl_3$

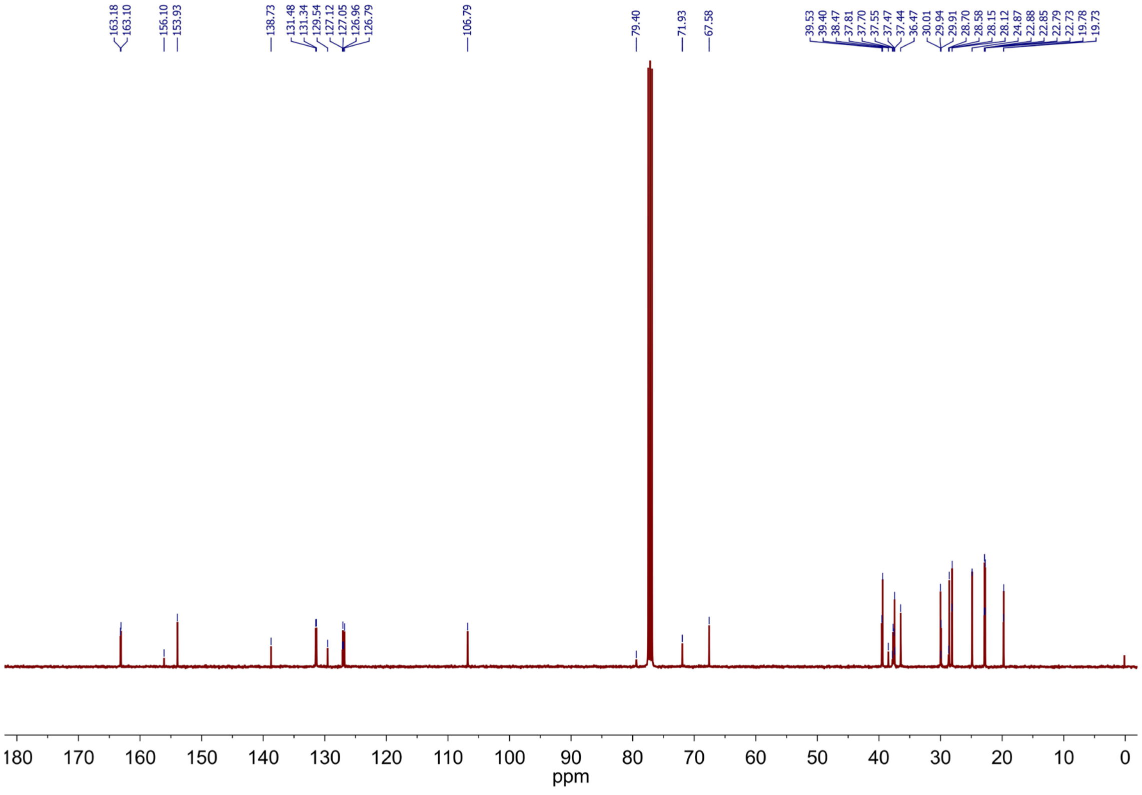

IR-FT spectrum of NDI-2 (solid state).

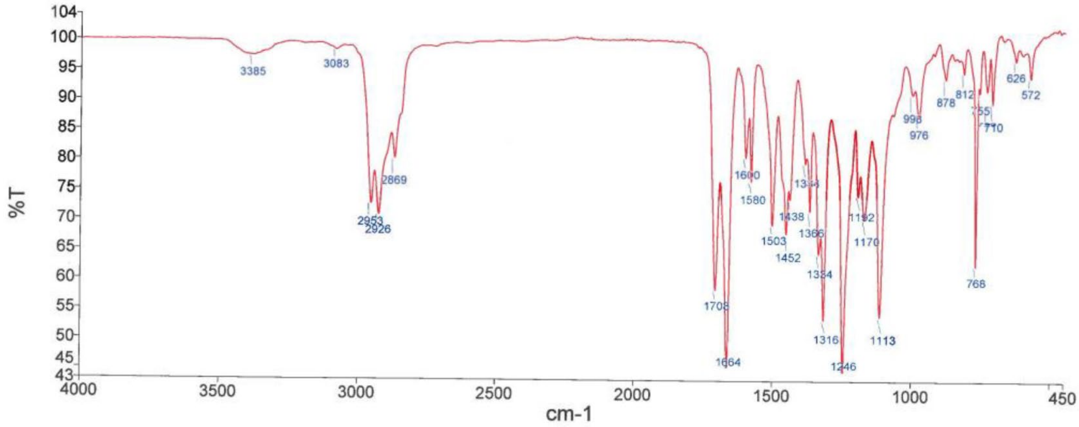

MALDI-TOF mass spectrum and isotopic pattern of NDI-1 in two different matrixes (CHCA and DCTB).

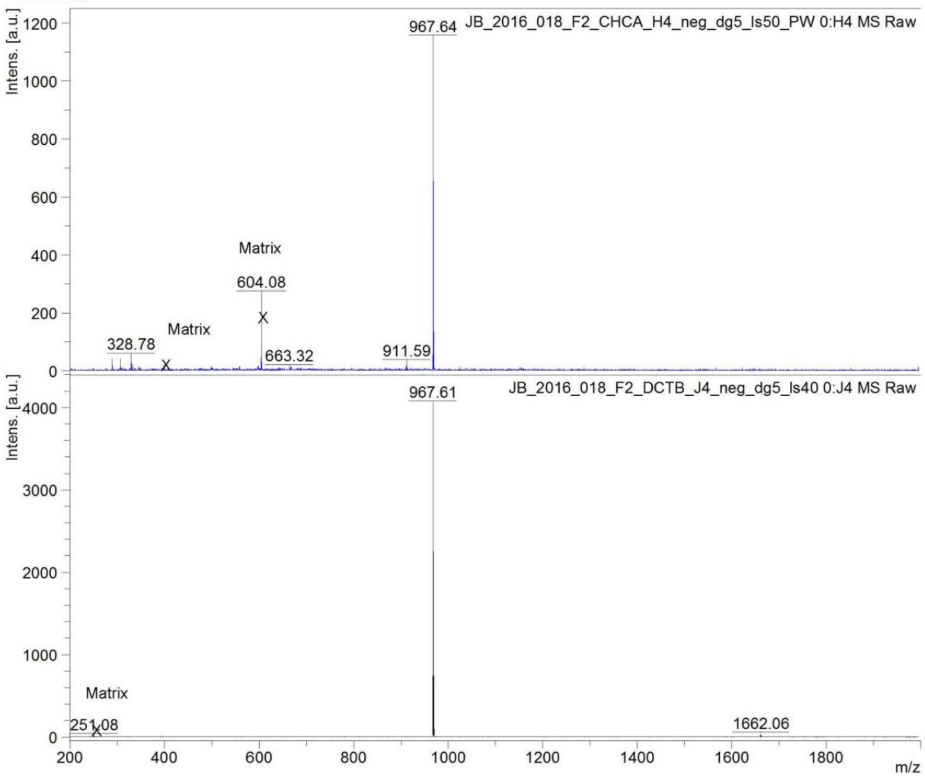

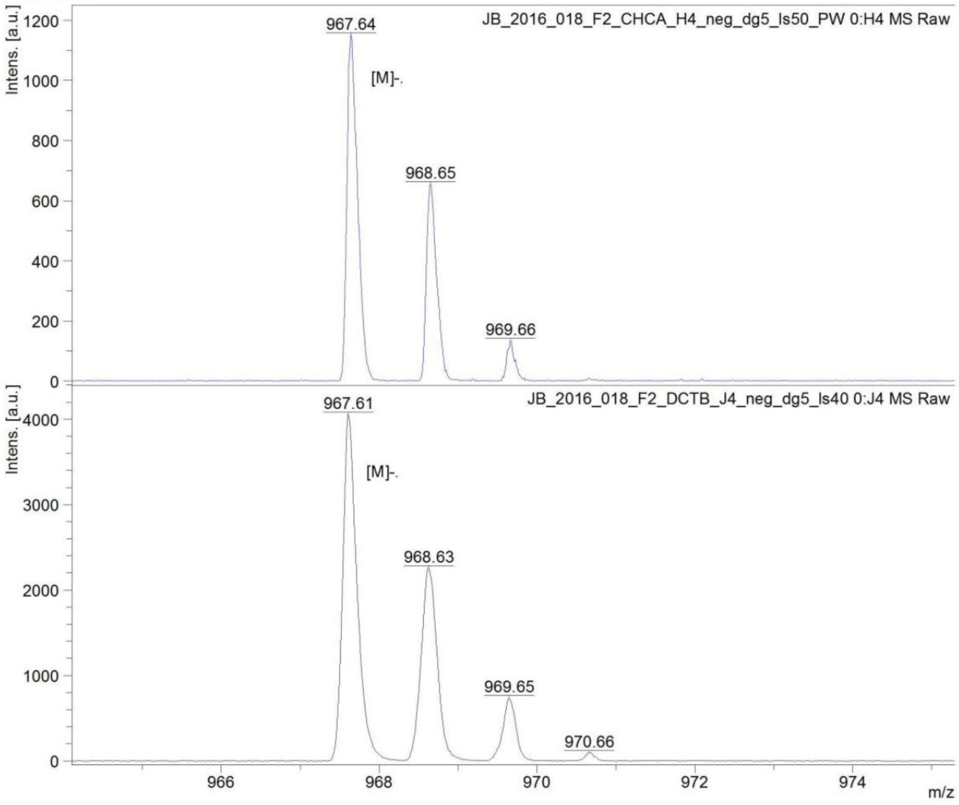

JB_2016_018_F2_CHCA_H4_neg_dg5_ls50_PW 0:H4 MS Raw
Intens. [a.u.]
967.64
[M]-.
968.65
969.66
JB_2016_018_F2_DCTB_J4_neg_dg5_ls40 0:J4 MS Raw
Intens. [a.u.]
967.61
[M]-.
968.63
969.65
970.66
m/z

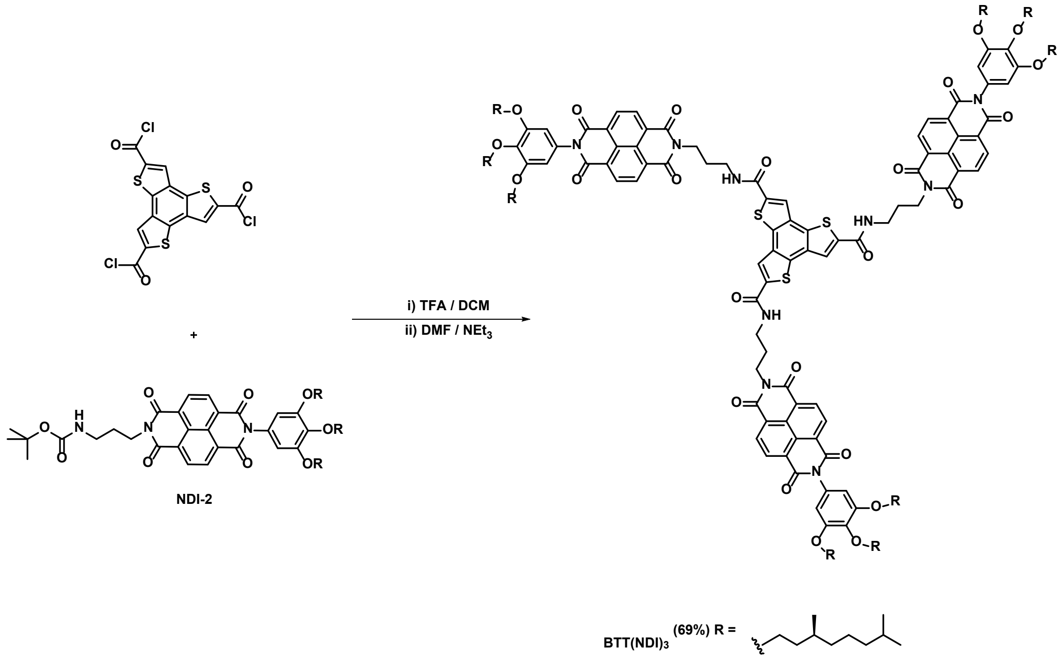

Scheme S2: Synthetic scheme of **BTT(NDI)3**.

Compound BTT(NDI)$_3$

In a 100 mL two-necked flask, dissolve **NDI-2** (0.31 g, 0.32 mmol) in 60 mL of DCM. Subsequently, 3.2 mL of TFA are added and the mixture is left to react for 3h. Once the reaction is complete, the solvent ant the TFA were removed under reduced pressure. Then, the crude was redissolved in dry THF (50 mL) and a suspension of BTT acid chloride[3] (0.030 g, 0.07 mmol) in dry THF (10 mL) and triethylamine (0.11 mL, 0.81 mmol) were added dropwise simultaneously. The mixture was refluxed for 15 h and the solvent was removed in vacuum. The solvent was removed under reduced pressure and the crude obtained was purified by column chromatography on silica gel using a Toluene/hepane mixture (2/1 v/v) and further recrystallized in the same mixture, to afford an orange crystalline solid (0.137 g, 58%).

^{1}H-NMR (400 MHz, $CDCl_3$) δ: 8.80 (m, 12H, NDI), 7.83 (s, 3H), 7.69 (s, 3H, NHCO), 6.51 (s, 6H, Ar), 4.40 (m, 6H, CH2-NDI), 4.11-4.04 (m, 18H, CH2-O), 3.60 (s, 6H, CH2-NH), 2.17 (s, 9H), 1.85-1.56 (m, 30H), 1.46-1.14 (m, 60H), 0.94-0.84 (m, 80H). **^{13}C-RMN** (101 MHz, CDCl3, δ) 163.05, 163.04, 161.84, 151.81, 139.94, 138.70, 135.34, 131.48, 131.33, 130.09, 128.74, 127.75, 127.01, 126.95, 126.69, 106.78, 71.82, 67.51, 39.41, 39.28, 37.56, 37.34, 36.35 29.89, 28.94, 29.91, 28.44, 27.99, 24.73, 22.69, 22.58, , 19,59. **FT-IR** (ATR) ν (cm^{-1}): 3270, 2952, 2926, 2869, 1708, 1664, 1600, 1580, 1503, 1452, 1384, 1366, 1334, 1316, 1246, 1192, 1170,1113, 767, 728, 626. **MS** MALDI-TOF (DCTB + NaI): *m/z* calc $C_{174}H_{231}N_9O_{24}S_3Na$: 2949.7 (100) $[M+Na]^+$; *m/z* found $C_{174}H_{231}N_9O_{24}S_3Na$: 2949.6 (100) $[M+Na]^+$, 2972.61 (10) $[M+2Na]^+$. **HR-MS** MALDI-TOF (DCTB NaI): *m/z* calc $C_{174}H_{231}N_9O_{24}S_3Na$: 2949.6186 $[M+Na]^+$; m/z found $C_{174}H_{231}N_9O_{24}S_3Na$: 2949.6168 $[M+Na]^+$.

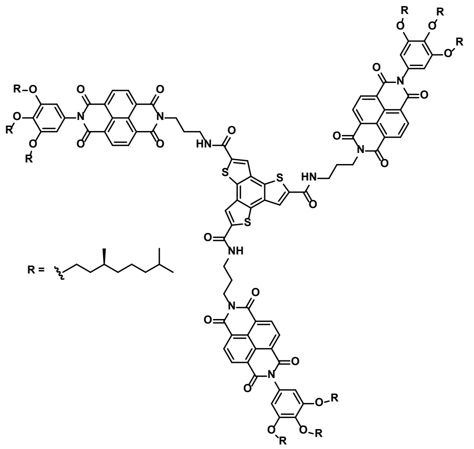

^{1}H NMR spectrum of BTT(NDI)$_3$ in $CDCl_3$

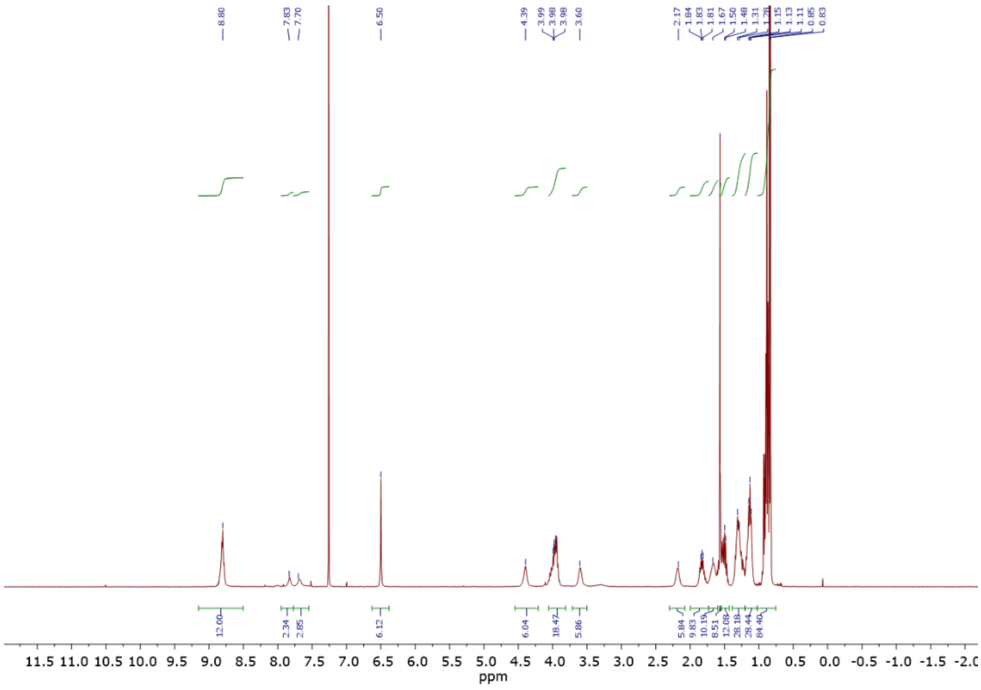

^{13}C NMR spectrum of BTT(NDI)$_3$ in $CDCl_3$

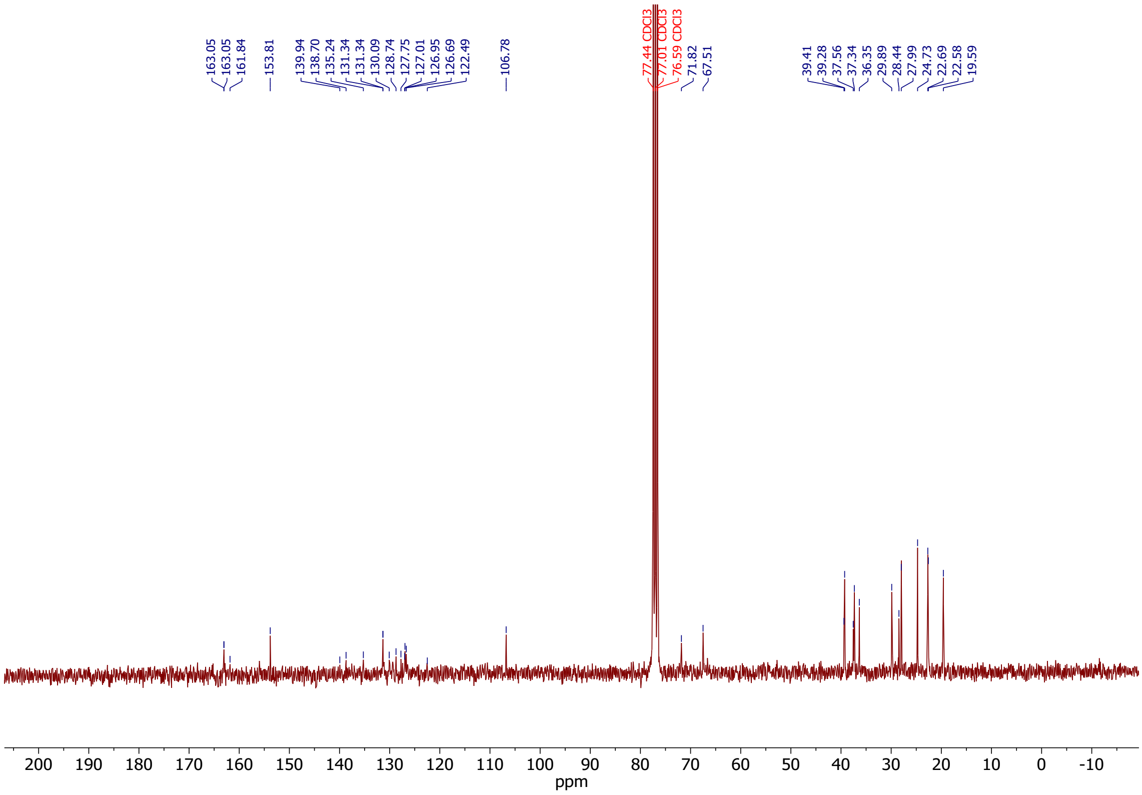

IR-FT spectrum of BTT(NDI)$_3$ (solid state).

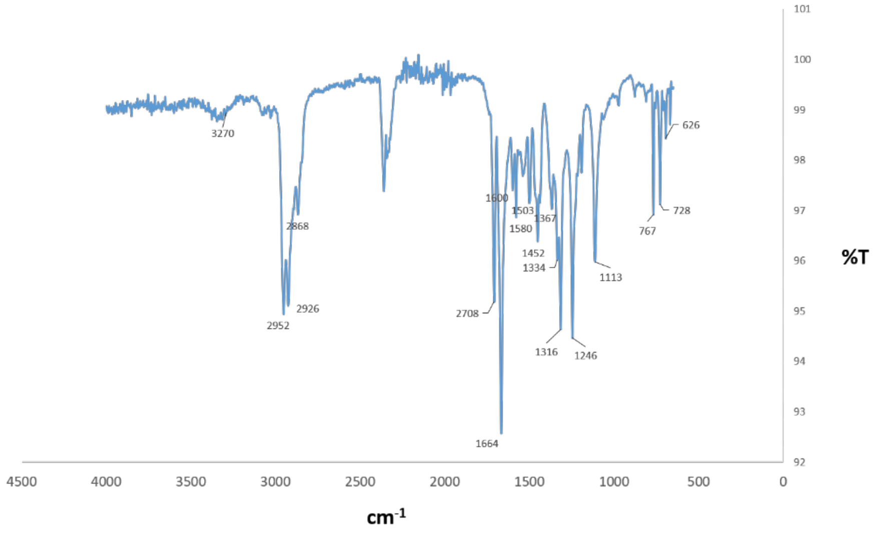

MALDI-TOF mass spectrum of BTT(NDI)$_3$ and experimental (top) and simulated (bottom) isotopic pattern of the molecular ion signal.

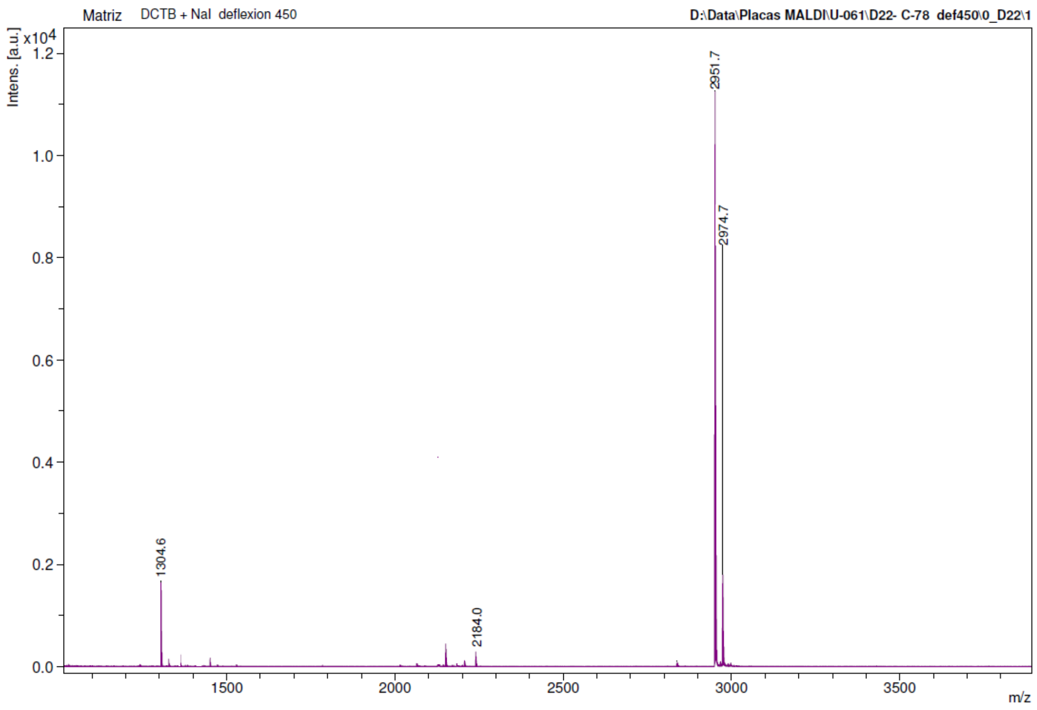

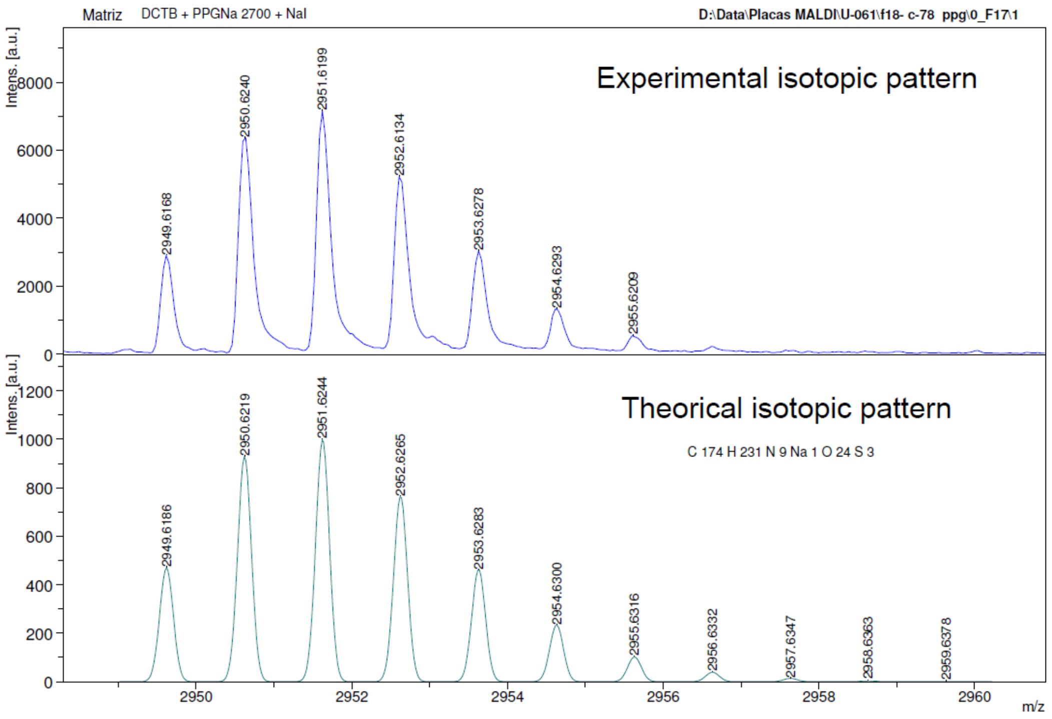

Supporting data

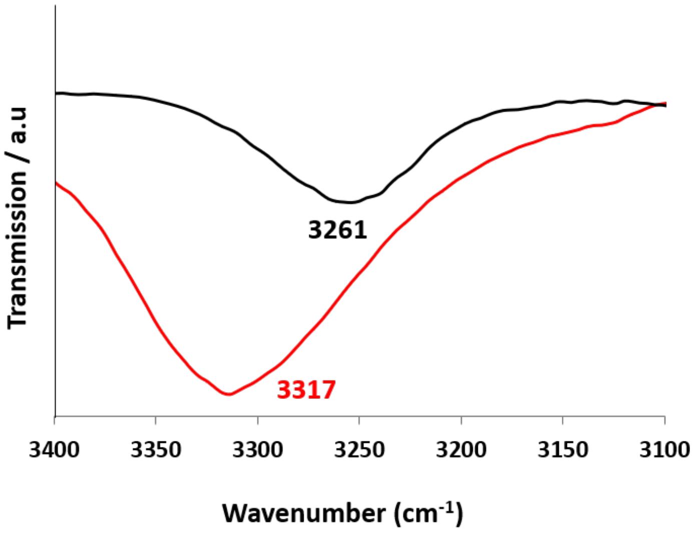

Fig. S1 Fourier-transform infra-red (FT-IR) spectra of compound **BTT(NDI)$_3$** dispersed in toluene (black) and THF (red). The N-H stretching demonstrates the formation of hydrogen bonds during self-assembly in toluene.

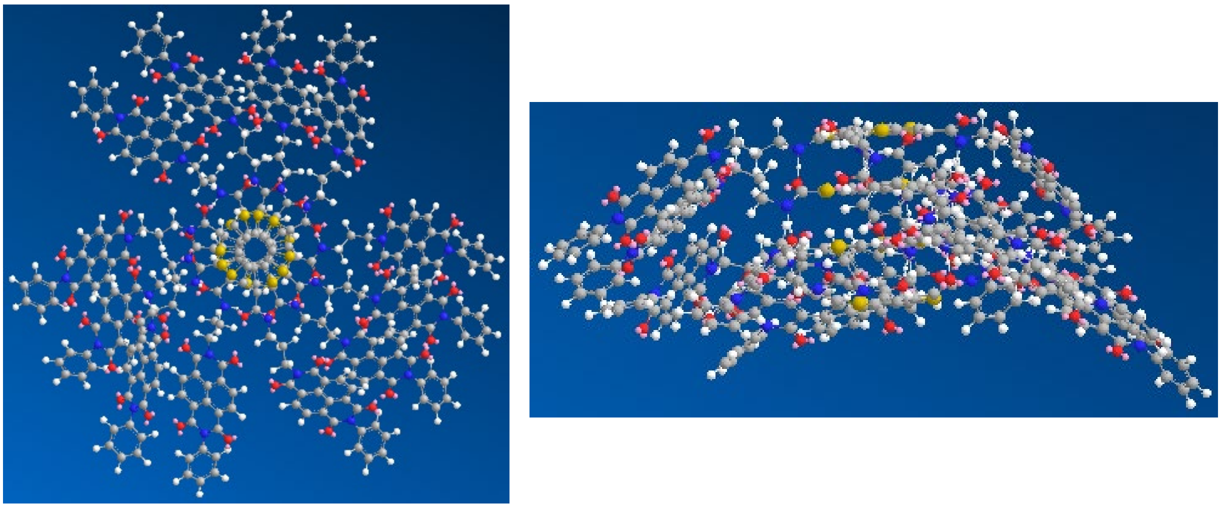

Fig. S2. Optimized (B3LYP/3-31G) structure of a tetrameric stack of **BTT(NDI)$_3$**, where peripheral alkoxy groups were removed in order to reduce the computational cost. All amide groups in the model rotate out of the thiophene plane by 40° to engage in hydrogen bonding interactions (N...H distance 1.81 A°). This creates a helical stack where each BTT unit rotate 23° respect to the previous one being the distance between them BTTs 3.40 A°. The proquiral nature of benzotrithiophene, either chiral (RRRR is shown in Figure 3) or racemic suppose a negligible difference in energy (<1 kcal/mol in favor of the racemic tetramer) as previously observed.[2] NDI plane to plane mutual distances are 3.55 A° showing a classical J-aggregate angle of 34°.

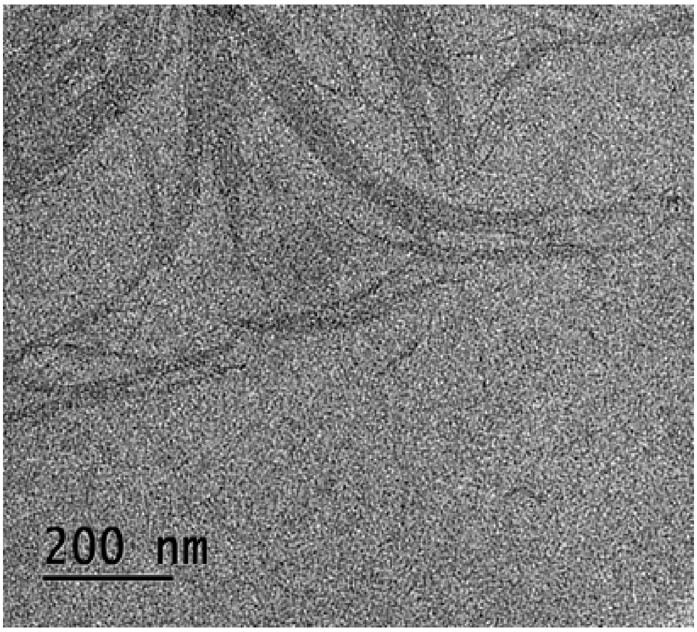

Fig. S3 TEM image of a sample of **BTT(NDI)$_3$** prepared from drop-cast toluene solution 2.5 x 10^{-6}M.

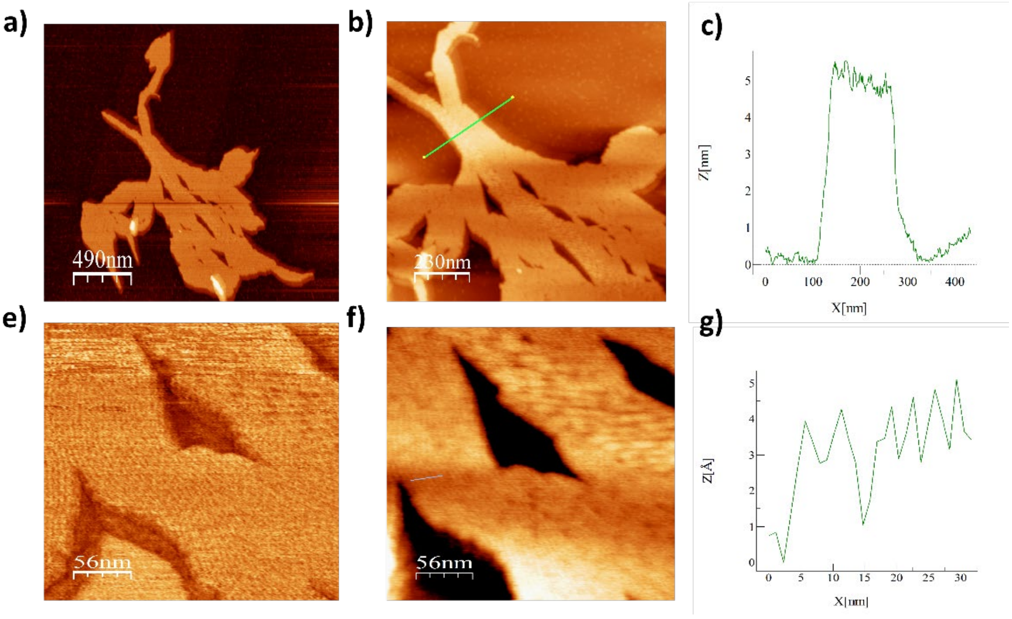

Fig. S4 AFM images (topographical scan a, b and c) and phase (e) of **BTT(NDI)$_3$** nanofiber bundles, prepared from a 1.5 x 10^{-6}M drop-cast toluene solution on HOPG. c) Height profile along the green line in b). g) Height profile along the grey line in f).

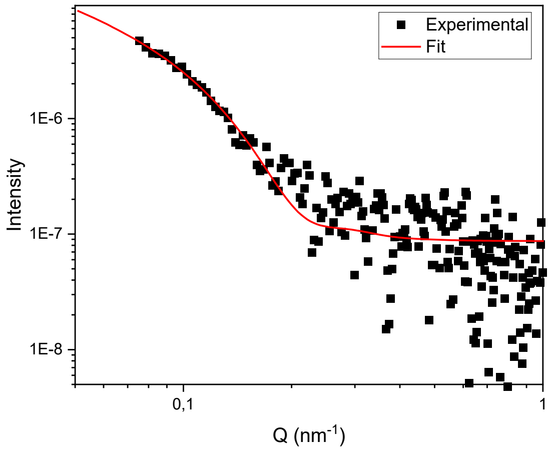

Fig. S5 SAXS profile of a 10^{-4} M toluene solution of **BTT(NDI)$_3$**. The experimental data are shown as black squares, while the red solid line is the fit to a cylindrical form factor.

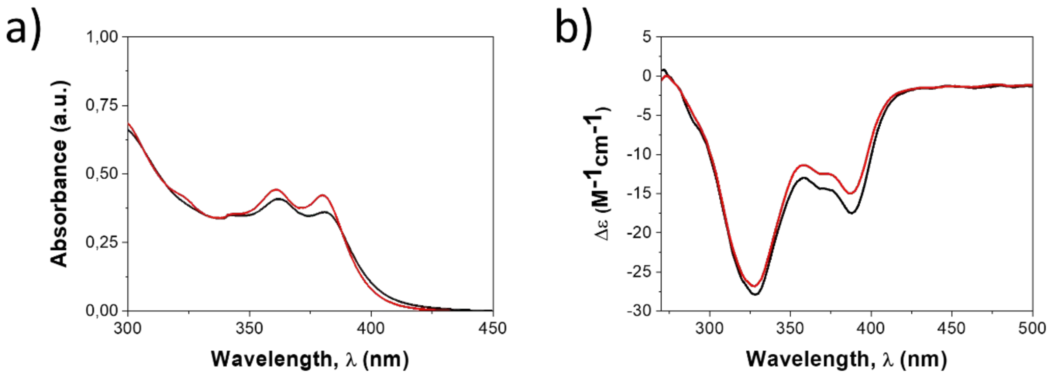

Fig. S6 a) UV-Vis and b) CD spectra of **BTT(NDI)$_3$** in toluene at 1.0 x 10^{-5} M at different temperatures between 298 K (black) and 370 K (red).

The Gibbs free energy of monomer association (ΔG), the parameter *m*, that relates the ability of the good solvent to interact with the monomer, and the cooperativity degree (σ) have been derived by using the SD model (Fig. 3d,e in the main text). The application of eqn (1) – (3) allows the derivation of the complete set of thermodynamic parameters associated with the supramolecular polymerisation mechanism of **BTT(NDI)$_3$**.

$$\Delta G' = \Delta G + mf \quad (1)$$

$$\Delta G' = -RTlnK_e \quad (2)$$

$$\sigma = K_n/K_e \quad (3)$$

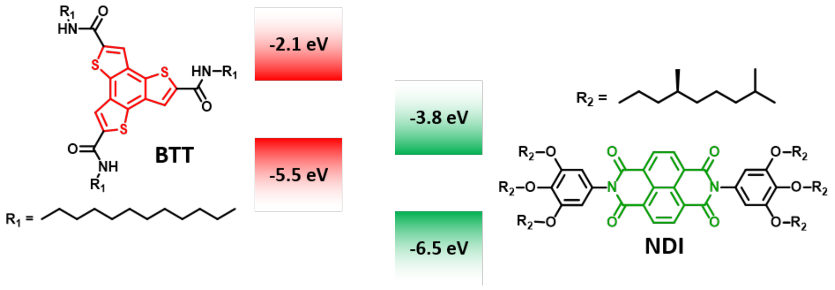

Figure S7. Energy diagram of BTT(NDI)$_3$ based on cyclic voltammetry experiments. (CV) of the individual components.

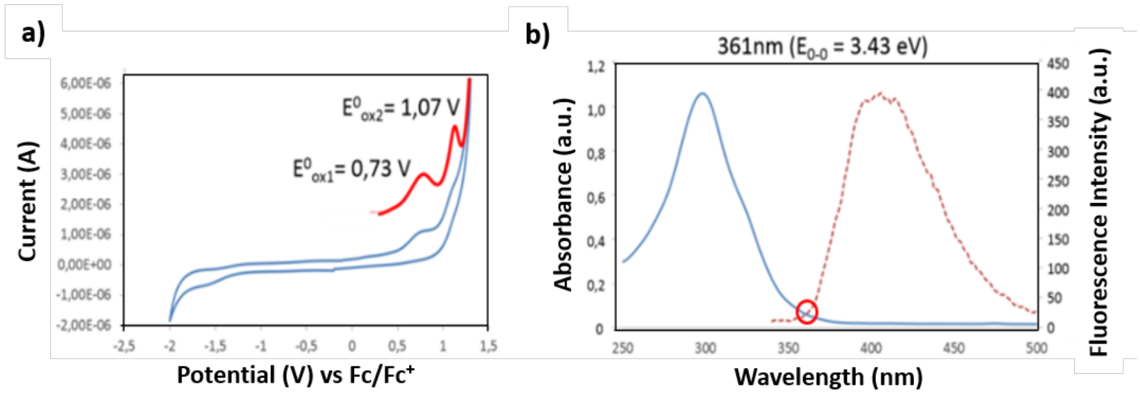

Figure S8. a) Cyclic voltammogram of **BTT**. Potential values are registered vs Ag/AgNO$_3$ reference electrode, inset: Square Wave Voltammetry (SWV) (red line); b) Absorption (solid line) and fluorescence spectra (dashed line) of BTTTA (excitation wavelength, 287 nm). The HOMO energy level was obtained using the approximation $E_{\mathrm{HOMO}} = -4.8 - E_{\mathrm{ox}}^{1/2}$ (vs Fc/Fc$^+$) = −5.5 eV. The LUMO energy level was obtained by using optical ($E_{0\text{-}0}$) bandgap obtained from the interception between the absorption and emission spectra $E_{\mathrm{LUMO}} = E_{\mathrm{HOMO}} + E_{0\text{-}0}$ = −2.1 eV

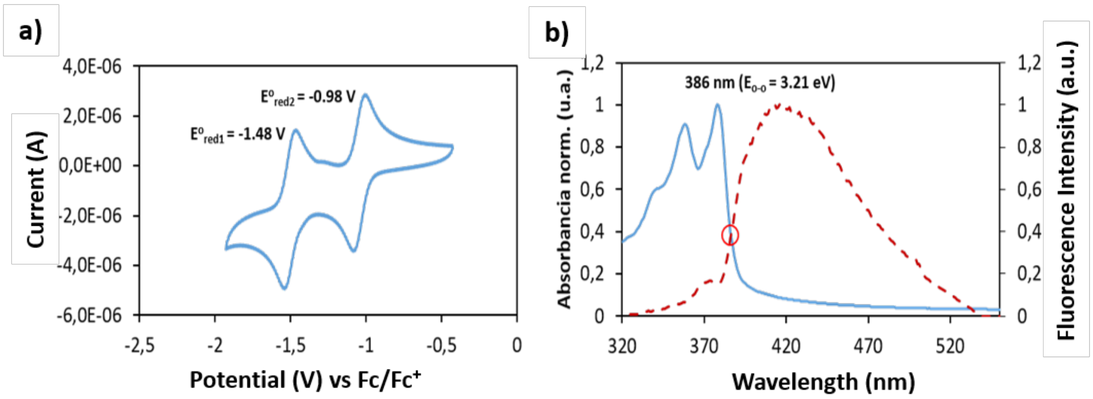

Figure S9. a) Cyclic voltammogram of NDI. Potential values are registered vs Ag/AgNO$_3$ reference electrode, b) Absorption (solid line) and fluorescence spectra (dashed line) of NDI (excitation wavelength, 300 nm). The HOMO energy level was obtained using the approximation $E_{\mathrm{LUMO}} = -4.8 - E_{\mathrm{red}}^{1/2}$ (vs Fc/Fc$^+$) = −3.8 eV. The homo energy level was obtained by using optical ($E_{0\text{-}0}$)

bandgap obtained from the interception between the absorption and emission spectra $E_{LUMO} = E_{HOMO} + E_{0-0} = -6.5$ eV.

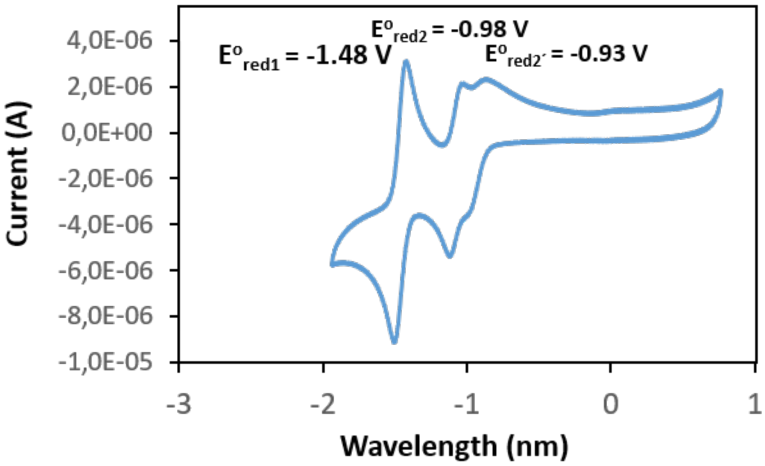

Figure S10. a) Cyclic voltammogram of **BTT(NDI)₃**. Potential values are registered vs Ag/$AgNO_3$ reference electrode. In **BTT(NDI)₃** cyclovoltammetry, only two reduction potentials (Ered1 and Ered2) and no oxidation potential (E_{ox}) were observed. With respect to **NDI**, the E_{red} values are very similar (Fig. S7)) except for a splitting observed in the first reduction potential (E_{red1} and $E_{red1'}$), something that can be associated with aggregation effects of the molecule. On the other hand, with respect to the **BTT**, no oxidation process was observed. This may be due to the presence of three NDI units that prevent the visibility of the processes and the difficulty that had already been encountered in the CV of **BTT**.

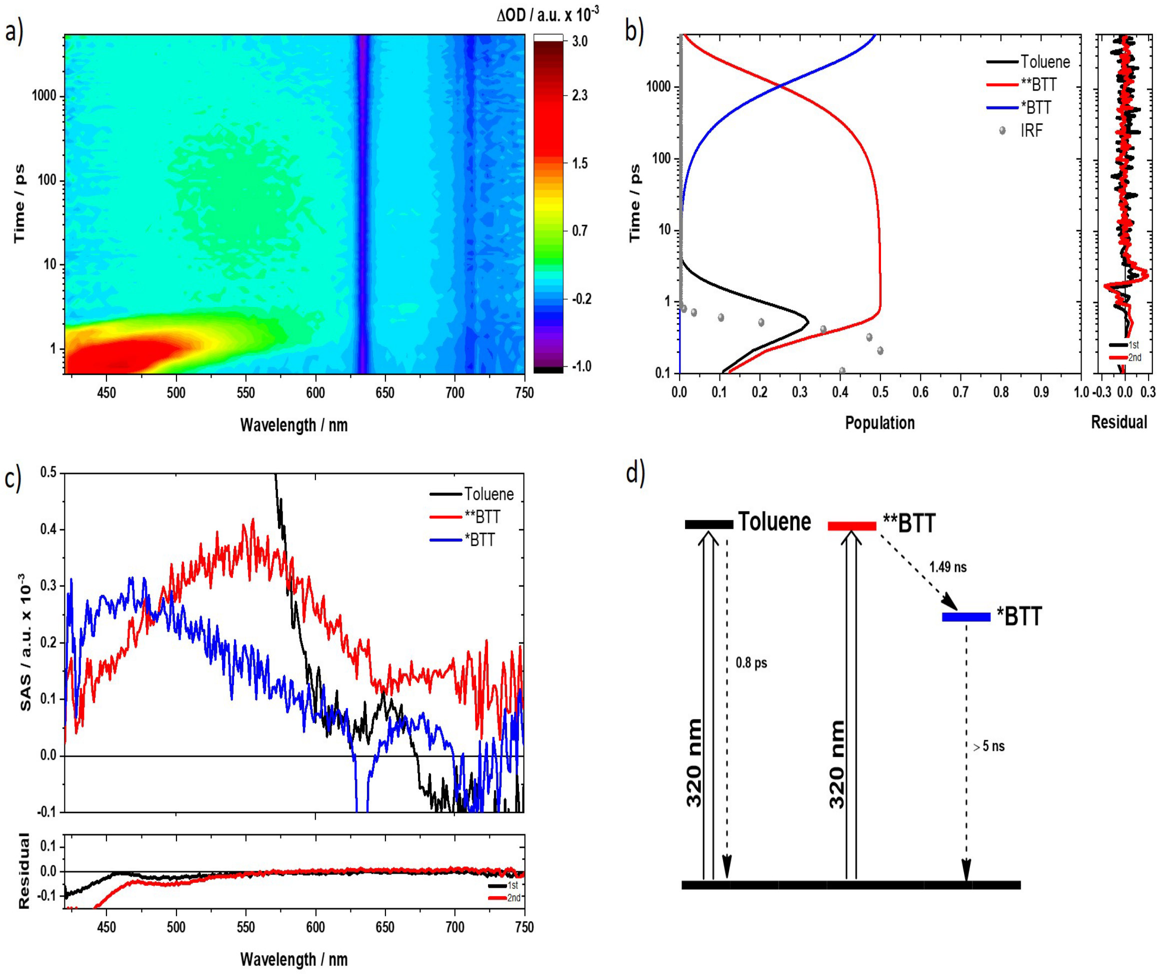

Figure S11. a) Differential absorption spectra obtained upon femtosecond pump-probe experiments (320 nm) of a solution containing **BTT** (1 x 10^{-5} M) in toluene with several time delays between 0.1 and 5500 ps. b) Population over time and c) SAS obtained upon chirp corrected GloTarAn analysis of the raw data with toluene (black), **BTT (red), *BTT (blue), IRF (grey dots), and corresponding first (black) and second (red) singular vectors of the residual on the right (for b) and bottom (for c). d) Mechanistic model applied in GloTarAn. Relative energies are arbitrary in

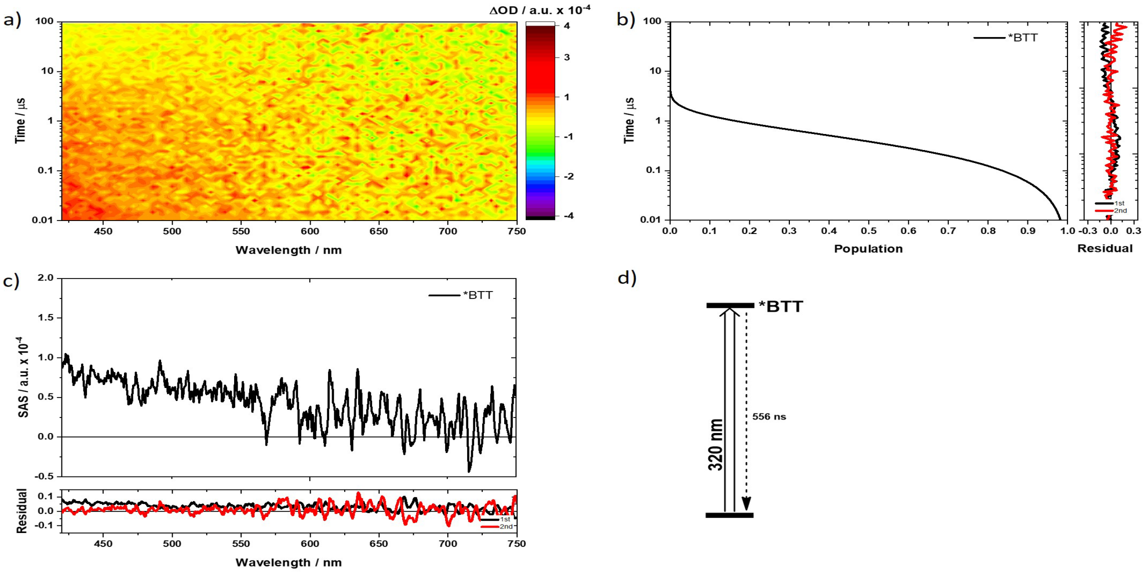

Figure S12. a) Differential absorption spectra obtained upon nanosecond pump-probe experiments (320 nm) of a solution containing **BTT** (1×10^{-5} M) in toluene with several time delays between 0.01 and 100 µs. b) Population over time and c) SAS obtained upon GloTarAn analysis of the raw data with *BTT (black) and corresponding first (black) and second (red) singular vectors of the residual on the right (for b) and bottom (for c). d) Mechanistic model applied in GloTarAn. Relative energies are arbitrary in favor of clarity.

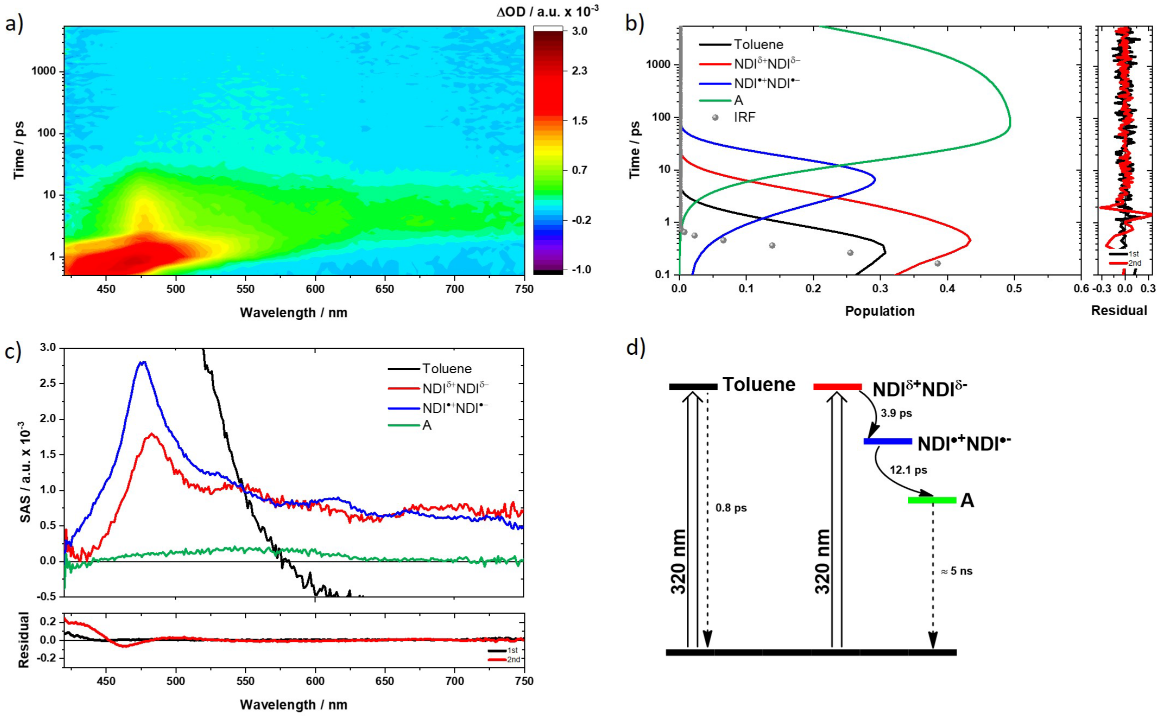

Figure S13. a) Differential absorption spectra obtained upon femtosecond pump-probe experiments (320 nm) of a solution containing **NDI** (1×10^{-5} M) in toluene with several time delays between 0.1 and 5500 ps. b) Population over time and c) SAS obtained upon chirp corrected GloTarAn analysis of the raw data with toluene (black), $NDI^{\delta+}NDI^{\delta-}$ (red), $NDI^{\bullet+}NDI^{\bullet-}$ (blue), product A (green), IRF (grey dots), and corresponding first (black) and second (red) singular vectors of the residual on the right (for b) and bottom (for c). d) Mechanistic model applied in GloTarAn. Relative energies are arbitrary in favor of clarity.

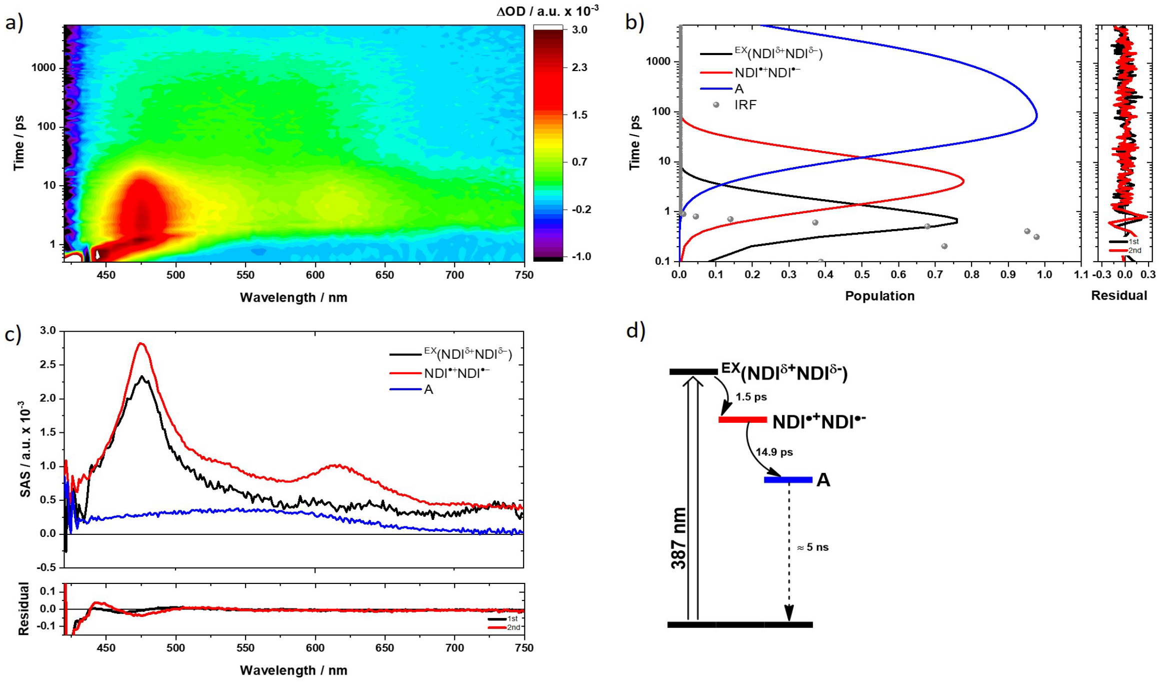

Figure S14. a) Differential absorption spectra obtained upon femtosecond pump-probe experiments (387 nm) of a solution containing **NDI** (c = 1 x 10^{-5} M) in toluene with several time delays between 0.1 and 5500 ps. b) Population over time and c) SAS obtained upon chirp corrected GloTarAn analysis of the raw data with $^{EX}(NDI^{\delta+}NDI^{\delta-})$ (black), $NDI^{\bullet+}NDI^{\bullet-}$ (red), product A (blue), IRF (grey dots), and corresponding first (black) and second (red) singular vectors of the residual on the right (for b) and bottom (for c). d) Mechanistic model applied in GloTarAn. Relative energies are arbitrary in favor of clarity.

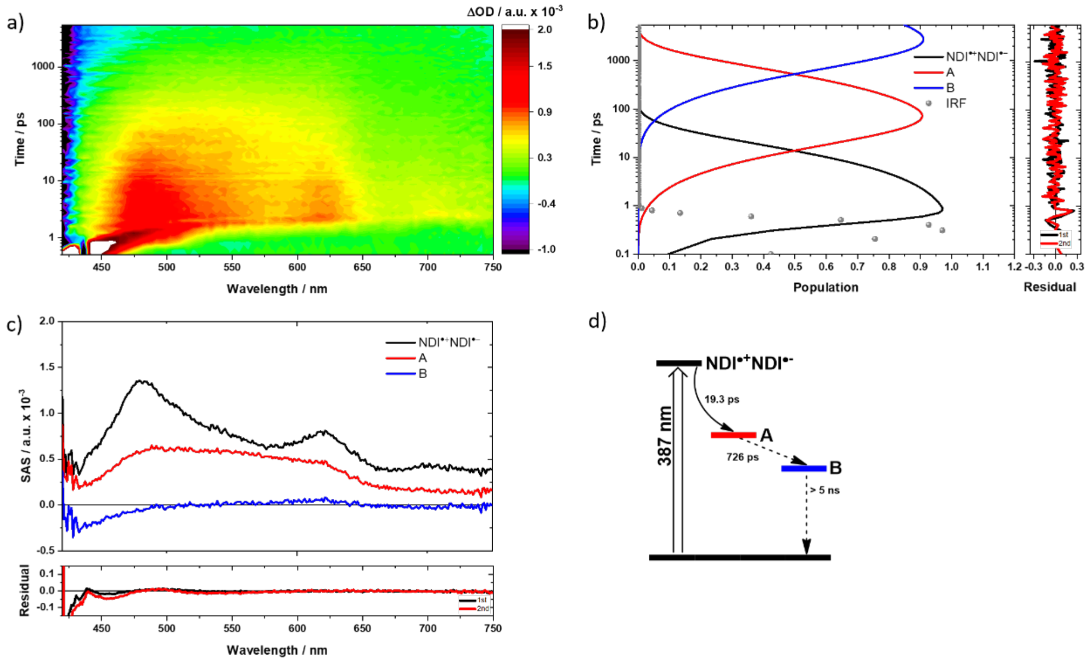

Figure S15. a) Differential absorption spectra obtained upon femtosecond pump-probe experiments (387 nm) of a solution containing **BTT(NDI)₃** (c = 2 x 10^{-5} M) in toluene with several time delays between 0.1 and 5500 ps. b) Population over time and c) SAS obtained upon chirp corrected GloTarAn analysis of the raw data with $NDI^{\bullet+}NDI^{\bullet-}$ (black), product A (red), product B (blue), IRF (grey dots), and corresponding first (black) and second (red) singular vectors of the residual on the right (for b) and bottom (for c). d) Mechanistic model applied in GloTarAn. Relative energies are arbitrary in favor of clarity.

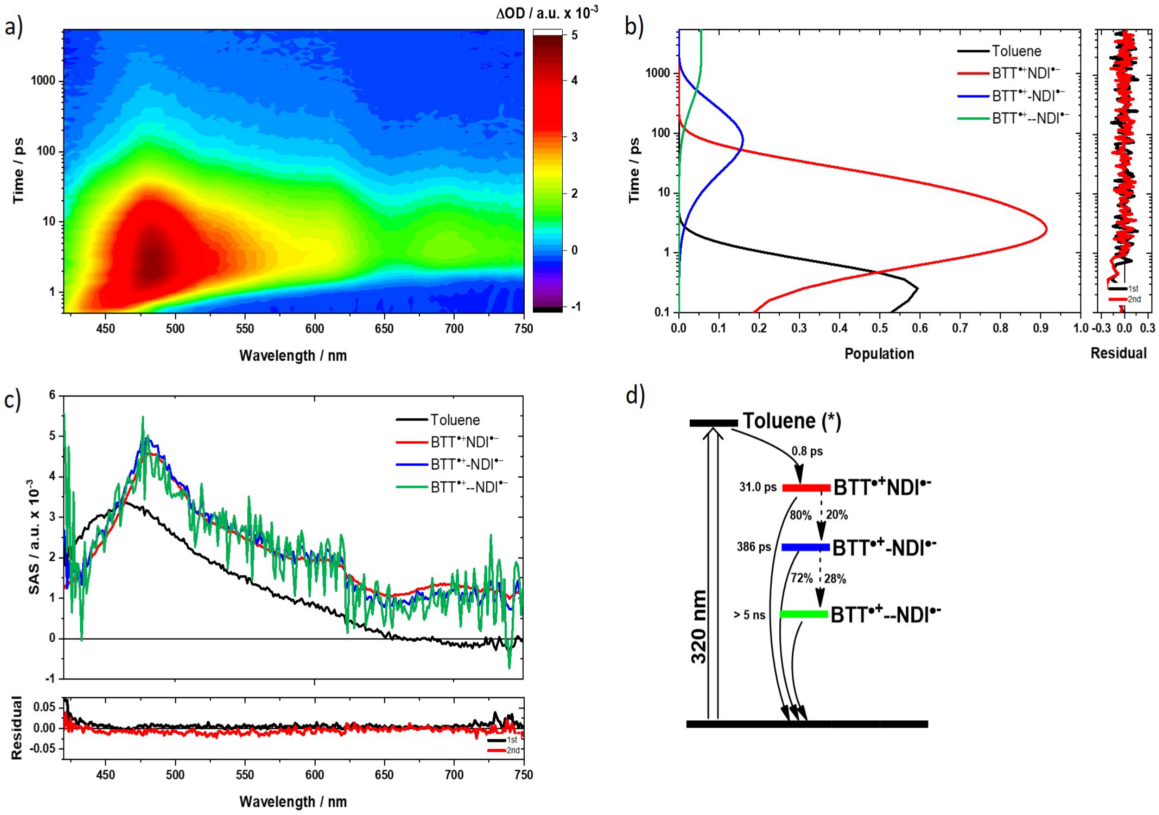

Figure S16. a) Differential absorption spectra obtained upon femtosecond pump-probe experiments (320 nm) of a solution containing **BTT(NDI)₃** (c = 2 x 10^{-5} M) in toluene with several time delays between 0.1 and 5500 ps. b) Population over time and c) SAS obtained upon chirp corrected GloTarAn analysis of the raw data with toluene (black), $BTT^{\bullet+}NDI^{\bullet-}$ (red), $BTT^{\bullet+}$-$NDI^{\bullet-}$ (blue), $BTT^{\bullet+}$--$NDI^{\bullet-}$ (green), IRF (grey dots), and corresponding first (black) and second (red) singular vectors of the residual on the right (for b) and bottom (for c). d) Mechanistic model applied in GloTarAn. Relative energies are arbitrary in favor of clarity. (*) It was not possible to separate the toluene decay from the $BTT^{\bullet+}$-$NDI^{\bullet-}$ pathway. Its absence did not change the overall model, but it was still included to achieve better spectral deconvolution of the early $BTT^{\bullet+}NDI^{\bullet-}$ species.

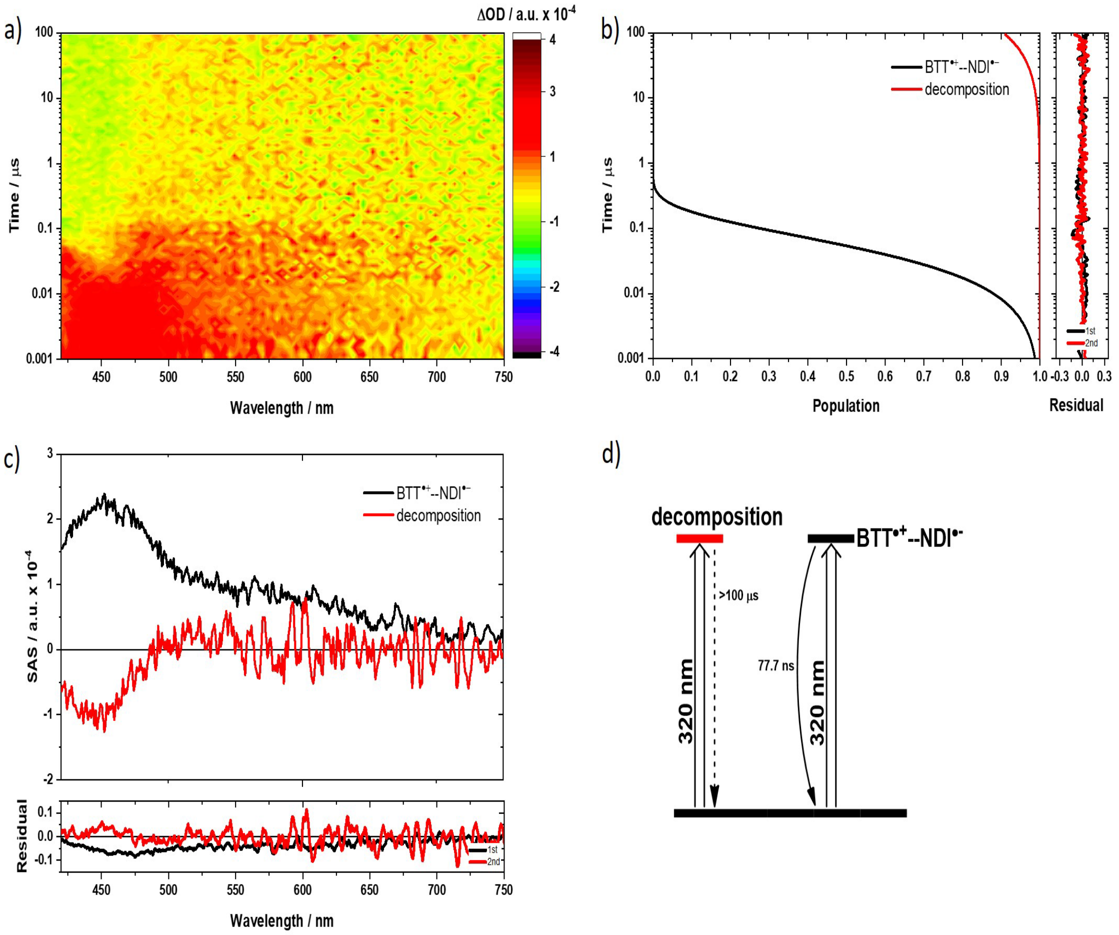

Figure S17. a) Differential absorption spectra obtained upon combined nanosecond pump-probe experiments (320 nm) of a solution containing **BTT(NDI)₃** (c = 2 x 10^{-5} M) in toluene with several time delays between 0.001 and 100 µs. b) Population over time and c) SAS obtained upon GloTarAn analysis of the raw data with $BTT^{•+}$--$NDI^{•-}$ (black), decomposition (red), and corresponding first (black) and second (red) singular vectors of the residual on the right (for b) and bottom (for c). d) Mechanistic model applied in GloTarAn. Relative energies are arbitrary in favor of clarity.

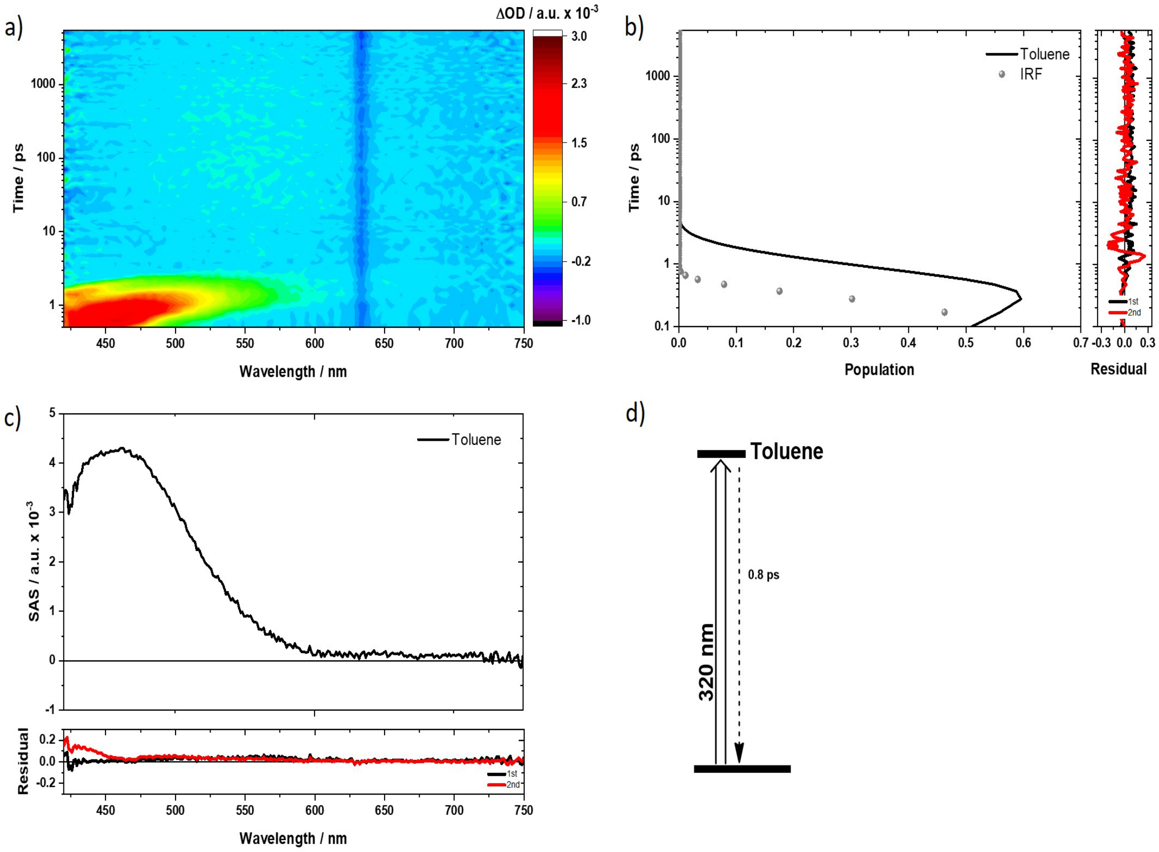

Figure S18. a) Differential absorption spectra obtained upon femtosecond pump-probe experiments (320 nm) of a toluene solution with several time delays between 0.1 and 5500 ps. b) Population over time and c) EAS obtained upon chirp corrected GloTarAn analysis of the raw data with toluene (black), IRF (grey dots), and corresponding first (black) and second (red) singular vectors of the residual on the right (for b) and bottom (for c). d) Mechanistic model applied in GloTarAn. Relative energies are arbitrary in favor of clarity.

References

1. a) van Stokkum, I. H. M.; Larsen, D. S.; van Grondelle, R., *BBA- Bioenergetics* **2004,** *1657*, 82-104; Snellenburg, J. J.; b) Laptenok, S.; Seger, R.; Mullen, K. M.; van Stokkum, I. H. M., Glotaran: *Journal of Statistical Software* **2012,** *49*, 1-22; c) Mullen, K. M.; van Stokkum, I. H. M., TIMP: An R Package for Modeling Multi-way Spectroscopic Measurements. *Journal of Statistical Software* **2007,** *18*, 1 - 46.

2. Percec, V; Aqad, E.; Peterca, M. Rudick, J. G.; Lemon, L.; Ronda, J. C.; De, B. B.; Heiney, P. A. Meijer, E. W. J. Am. Chem. Soc. 2006, 128, 16365-16372.

3. Demenev, A.; Eichhorn, S. H.; Taerum, T.; Perepichka, D. F.; Patwardhan, S.; Grozema, F.C.; Siebbeles, L. D. A.; Klenkler, R. *Chem. Mater.* **2010**, *22*, 1420–1428.
